# The earliest farming communities north of the Carpathians: The settlement at Gwoździec site 2

**DOI:** 10.1371/journal.pone.0227008

**Published:** 2020-01-15

**Authors:** Agnieszka Czekaj-Zastawny, Anna Rauba-Bukowska, Agnieszka Kukułka, Bernadeta Kufel-Diakowska, Maria Lityńska-Zając, Magdalena Moskal-del Hoyo, Jarosław Wilczyński

**Affiliations:** 1 Institute of Archaeology and Ethnology, Polish Academy of Sciences (IAE PAS), Kraków, Poland; 2 Regional Museum Tarnów, Tarnów, Poland; 3 Institute of Archaeology, University of Wrocław, Wrocław, Poland; 4 W. Szafer Institute of Botany, Polish Academy of Sciences (IB PAS), Kraków, Poland; 5 Institute of Systematics and Evolution of Animals, Polish Academy of Sciences (ISEA PAS), Kraków, Poland; University at Buffalo - The State University of New York, UNITED STATES

## Abstract

The appearance of the Linear Pottery Culture (LBK) on Poland territory initiated the process of neolithization in the area. However, as we will see in this article, this colonization took place later than previously thought. The stage, which in Poland is called as the early phase, actually corresponds only to the Fomborn/Ačkovy stage of LBK, and the earliest dating currently indicates around 5350 BC. Due to the small number of sites from this phase excavated on a large scale in Poland, this stage of the culture’s development is poorly known. The Gwoździec Project is focused on the earliest stage of LBK settlement in south-eastern Poland. Excavation at the site was finished in 2018. Therefore, the article presents preliminary results of interdisciplinary analyzes, such as research on ceramics, flint production and use, and botanical remains. They point to various aspects of the economy of these early agricultural communities and significantly enrich the knowledge of this period in Central Europe. They also expose the chronological development of the oldest LBK development stage in Poland.

## Introduction

**Fig 1:**
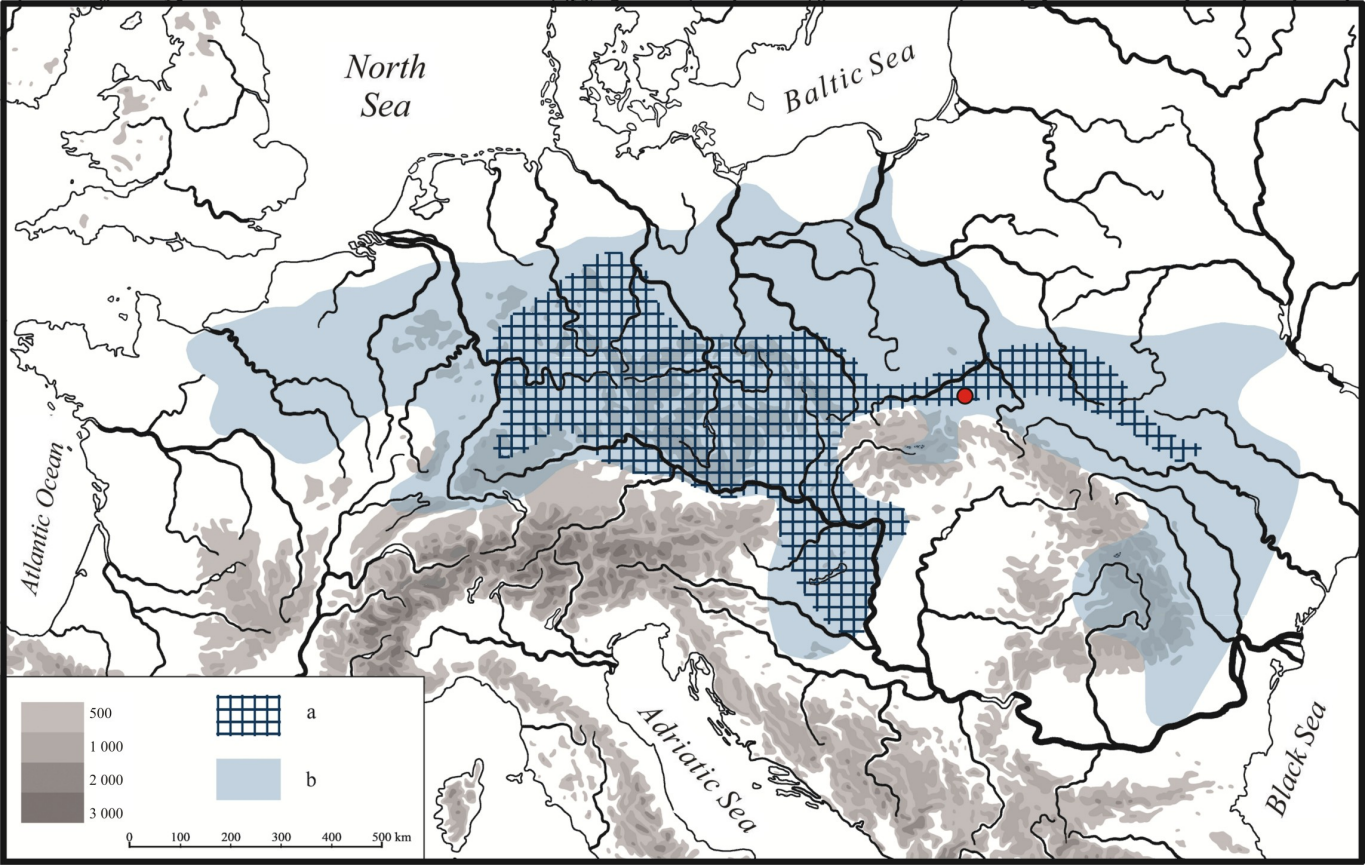
Linear pottery culture in Europe. Extent during the earliest phase (a), maximum extent (b) and the location of the Gwoździec site (red dot).

The appearance of a Linear Pottery Culture on Poland area initiated the process of neolithization in this area. This was a very important turning point, not only for the prehistory of this particular region, but for the entirety of Central Europe as well. Areas of the Upper Vistula River basin played a very important role in this respect, since this was the region embraced by the first stage of LBK expansion (Fig 1). The oldest stage in the area of our interest, sholud corresponds with the Bíňa-Bicske LBK phase (according to the Hungarian/Slovakian LBK sequence), and its beginnings seem to be dated to ca. 5500/5400 BC. However, as will be shown in this article, the first permanent settlements are only associated with the pre-Music Note stage, wchich is correlated to the Flomborn phase (according to German periodization) and the Ačkovy phase (Czech and Moravia sequence) [1]. Hence such assemblages in Poland are named as the early phase of the LBK, although in relation to the indigenous regions of this culture this is yet another development phase. The LBK in Poland is divided into three chronological and stylistic phases proposed by A. Kulczycka-Leciejewiczowa [2]. The first of them (LBK I) is divided into two subphases: Gniechowice (Ia) and Zofipole (Ib), although their materials are very often presented together simply as the early LBK [3].

The current project is focused on the earliest stage of LBK settlement in Lesser Poland. It will carry out analyses of settlement distribution by obtaining new evidence on the inner structures and functions of domestic sites, examining economic issues, and analyzing artefacts and anthropological data in order to determine absolute chronology. Taken together, these procedures should create the basis for reconstructing the processes accompanying the settlement of the first farmers, including the question of their origins, inter-regional contacts, and also the chronological frames of the early phase of the LBK in Lesser Poland, in correlation with neighbouring territories (occupied by other LBK groups and groups of the Eastern Linear Circle developing south of the Carpathian Mountains) [4].

Very intensive settlement by the LBK developed in the area of the Upper Vistula River basin; today some 800 settlement points dated to LBK phase II and III have been identified in this region [5, 6]. However, no more than 30 sites of the early LBK stage (phase I) are known from all over Poland. One of the most important is Gwoździec, com. Zakliczyn, in south-eastern Poland. This site will play a key role in the project. It is known from earlier test trenches. The newest (2016–2018) large-scale excavations are in progress. This paper presents the first results of this field research.

The site in Gwoździec is one of two settlements in Poland dated to the I phase of the LBK’s development that has been excavated on a large scale (the second is the site 12/13 in Targowisko [7]) Therefore, it can be helpful in revealing issues unknown to date, such as origins, economy, settlement spatial management, and dating the beginnings of this cultural unit. This site is unique in respect of its localization. It lies in a sub-mountainous area that was rarely chosen for establishing Early Neolithic settlements. The site was clearly attractive in many aspects, such as its location between the flint outcrops of the Kraków-Częstochowa Jura and the settlement range of the Eastern Linear Circle, with whom steady relationships were maintained.

## Materials and methods

The Gwoździec site is located in southern Poland, within the Carpathians flysch. It occupies an area of 2 hectares on loess subsoil. It is situated on the slope of a hump with culmination point reaching up to 329 m.a.s.l. and southern exposition. The slope descends towards the wooded valley of a small creek. The site was discovered in 1989 during a field survey. In 1996–2001 and 2006 minor excavations were carried out, during which the presence of features from the old LBK phase was confirmed [8, 9, 10].

**Fig 2:**
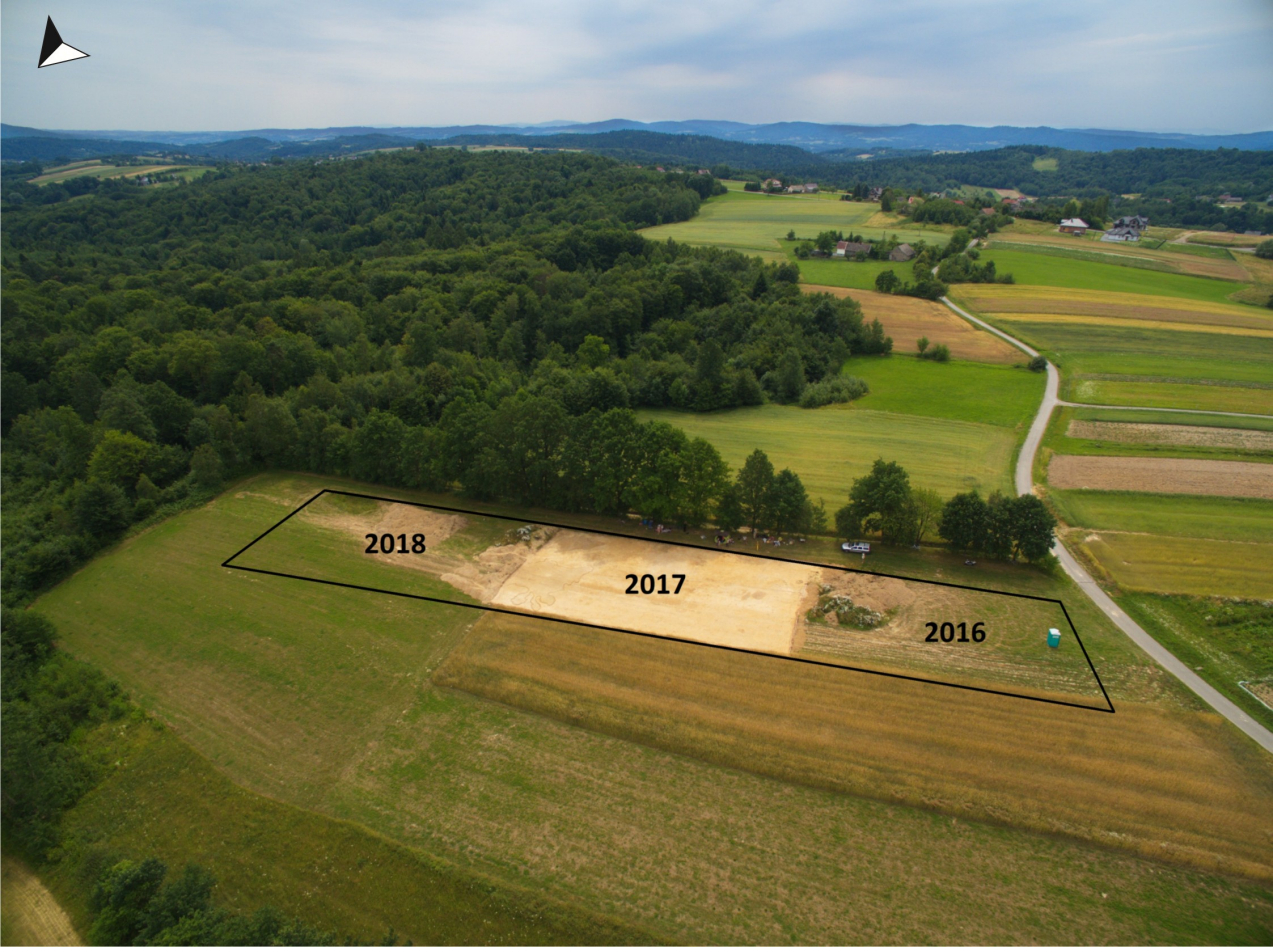
Gwoździec 2, Zakliczyn distr. **Location of trenches over subsequent seasons of research**.

Due to the encouraging results of the initial excavations, the site in Gwoździec became the main objective of the project mentioned in the Introduction. Within this project widespread investigations were performed in 2016–2018 (Fig 2). The excavations covered an area of ca. 30 ares. 164 settlement features were discovered. All of the pits identified in terms of their chronology belonged to the LBK. Along with the exploration, 3D documentation was drawn for all features, layers, and artefacts, while spatial analysis was performed using the GIS (Geographic Information System). All fillings of the explored features were subject to wet or dry sieving, and hundreds of samples were systematically gathered for specialized interdisciplinary analyses. Due to the fact that field research was completed less than a year ago, the interdisciplinary analyses are still underway. This paper presents the preliminary results of pottery and flint studies as well as archaeobotanical and chronological analyses. The chemical investigations on the ceramics (lipids) have not been completed. The full set of results will be addressed in the monograph dedicated to this site and subsequent articles.

No permits were required for the study described, which complied with all relevant regulations.

### Settlement features

**Fig 3:**
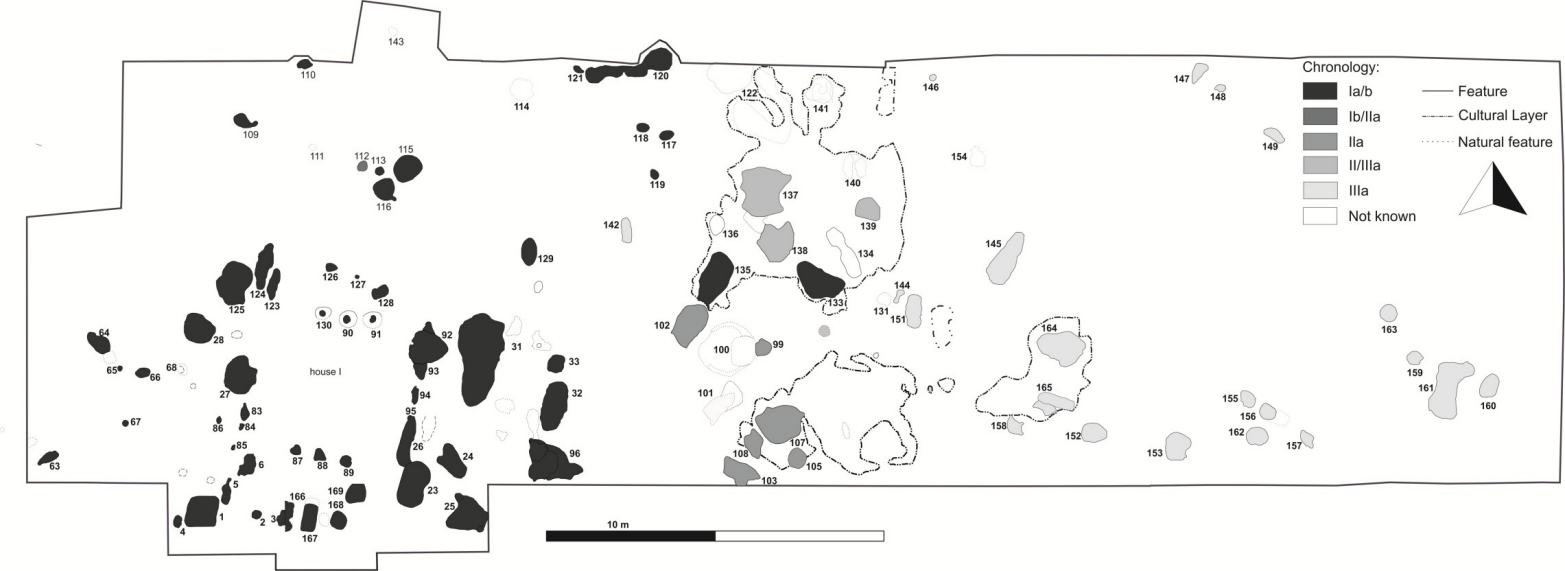
Gwoździec 2, Zakliczyn distr. **Layout of LBK settlement features**.

**Fig 4:**
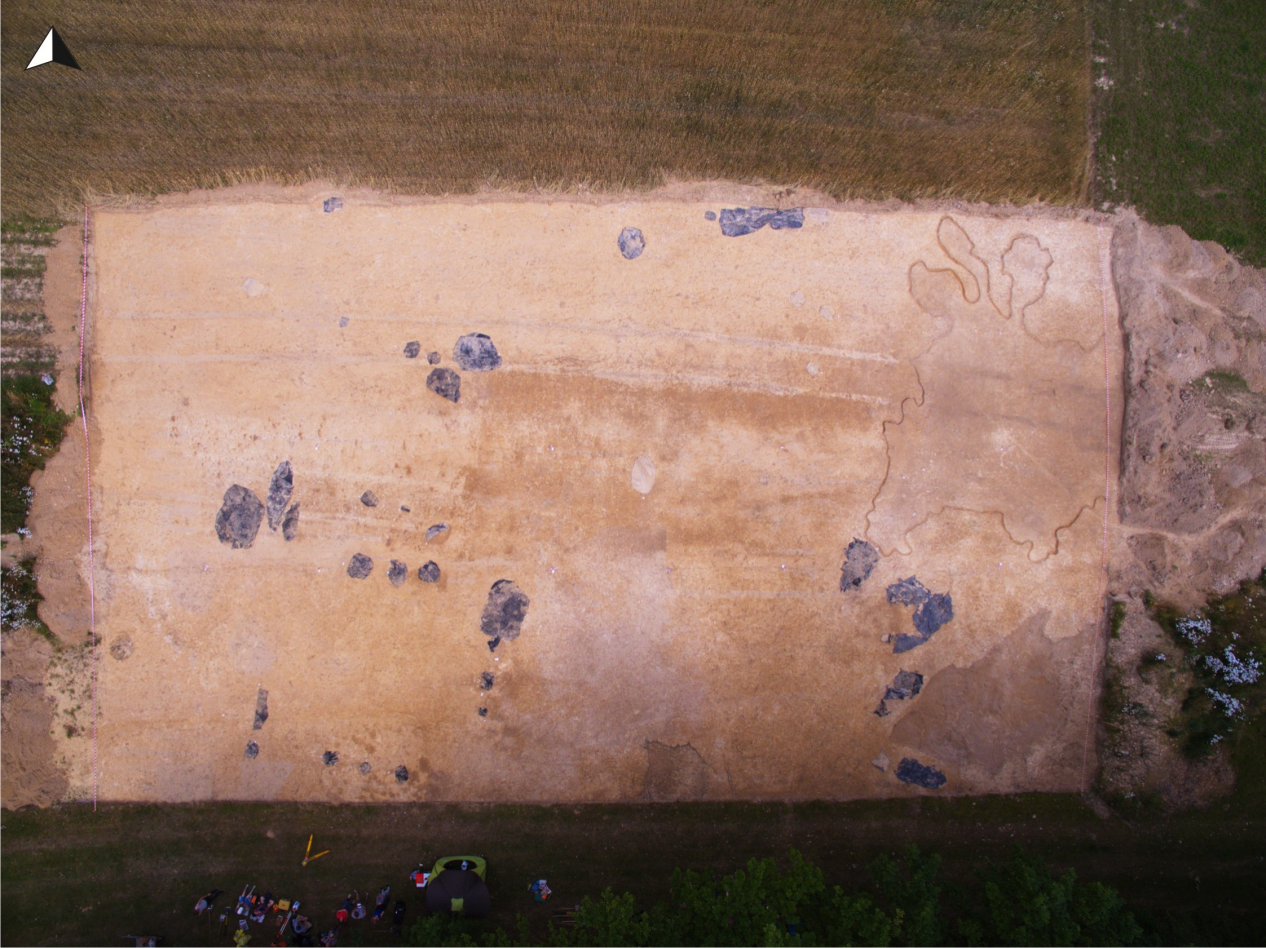
Gwoździec 2, Zakliczyn distr. **Aerial photo of settlement features from the early LBK phase**.

**Fig 5:**
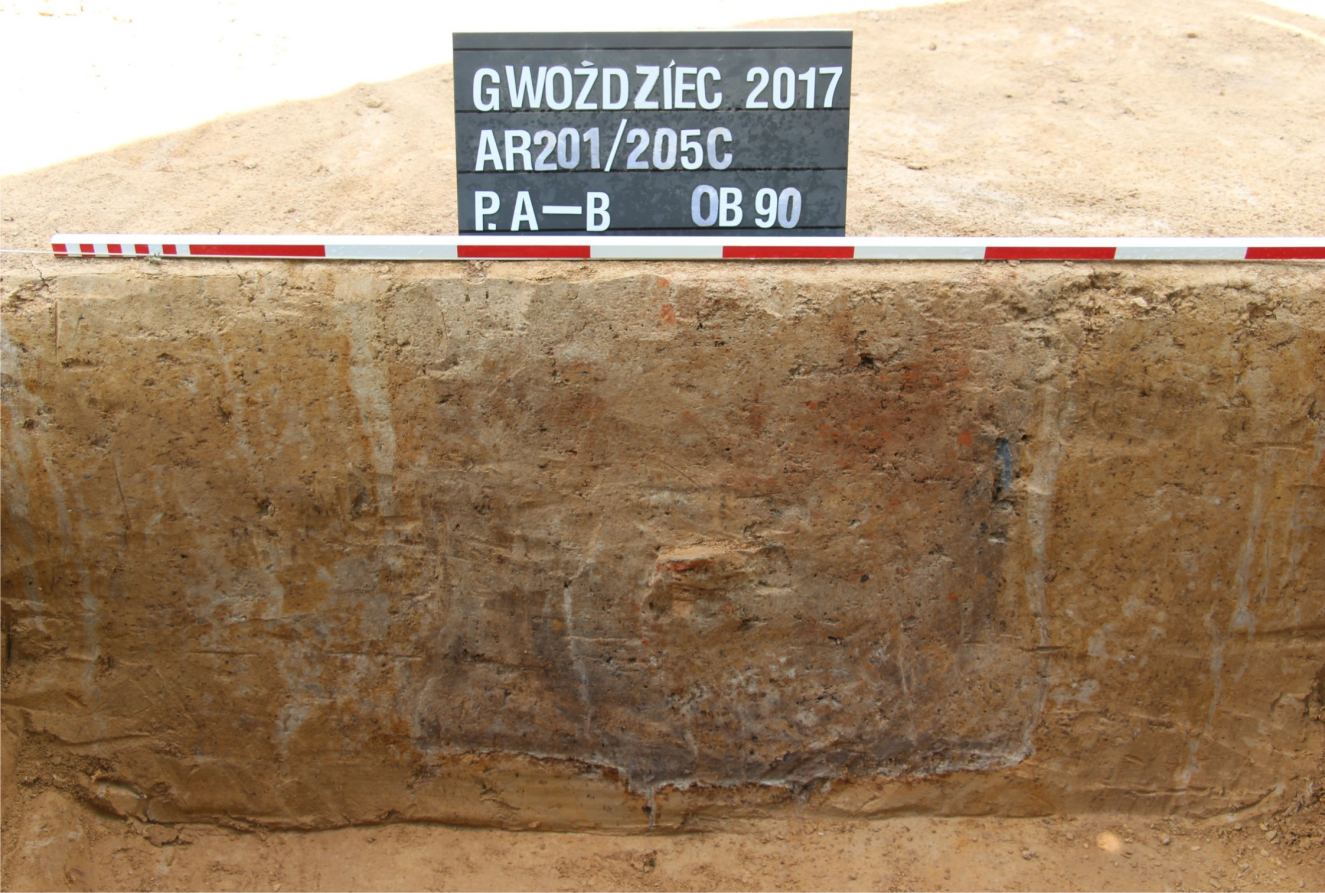
Gwoździec 2, Zakliczyn distr. **Profile of a posthole from the house**.

The most important discovery made at the site is that of the remains of a building complex from the early phase of LBK development (Fig 3). It consisted of a post-frame house and elongated complexes of pits that originally ran along its walls (Fig 4). The house construction has been preserved in the form of traces of three internal rows of supporting posts. These were massive posts made of trunks of oak (*Quercus*) and ash (*Fraxinus excelsior*), about 40–60 cm in diameter and preserved at a depth of up to ca. 75 cm (Fig 5). There are no traces of posts of external walls. Along the latter, on the other hand, elongated pits are located, and these are thought to have served as drainage ditches for rainwater in this type of house construction, due to their location beneath the roof eaves. Originally the house had a length of ca. 23 m and was ca. 6 m wide. It was closely surrounded by various utility pits, such as storage pits, garbage pits, and hearths. One of the most interesting is feature no. 1. This is a rectangular storage pit containing a large amount of pottery, flint, and charred remains of cereals. Another extraordinary find is a cultural layer that initially constituted the ground level of the settlement, and extended to the east from the building complex. Sediments of this kind are very rarely recorded. They are quite shallow and very susceptible to damage by modern cultivation of crop fields. This layer spread over a total area of about 150 m^2^ and reached a depth of 10–15 cm. It contained numerous fragments of vessels and flint artefacts.

To the east of the building complex more settlement features were identified, partly dug into the cultural layer mentioned above. Their fillings contained fragments of vessels identical in terms of stylistics to the pottery obtained from the area of the building complex, as well as with ceramics decorated with a small amount of ornamental motifs corresponding with the successive LBK phases (see Fig 3).

Within the fillings of features from the early LBK phase there are mostly fragments of pottery, flint, and stone artefacts, and abundant botanical remains. Unfortunately, the soil type was responsible for the total decomposition of faunal remains.

### Pottery

**Fig 6:**
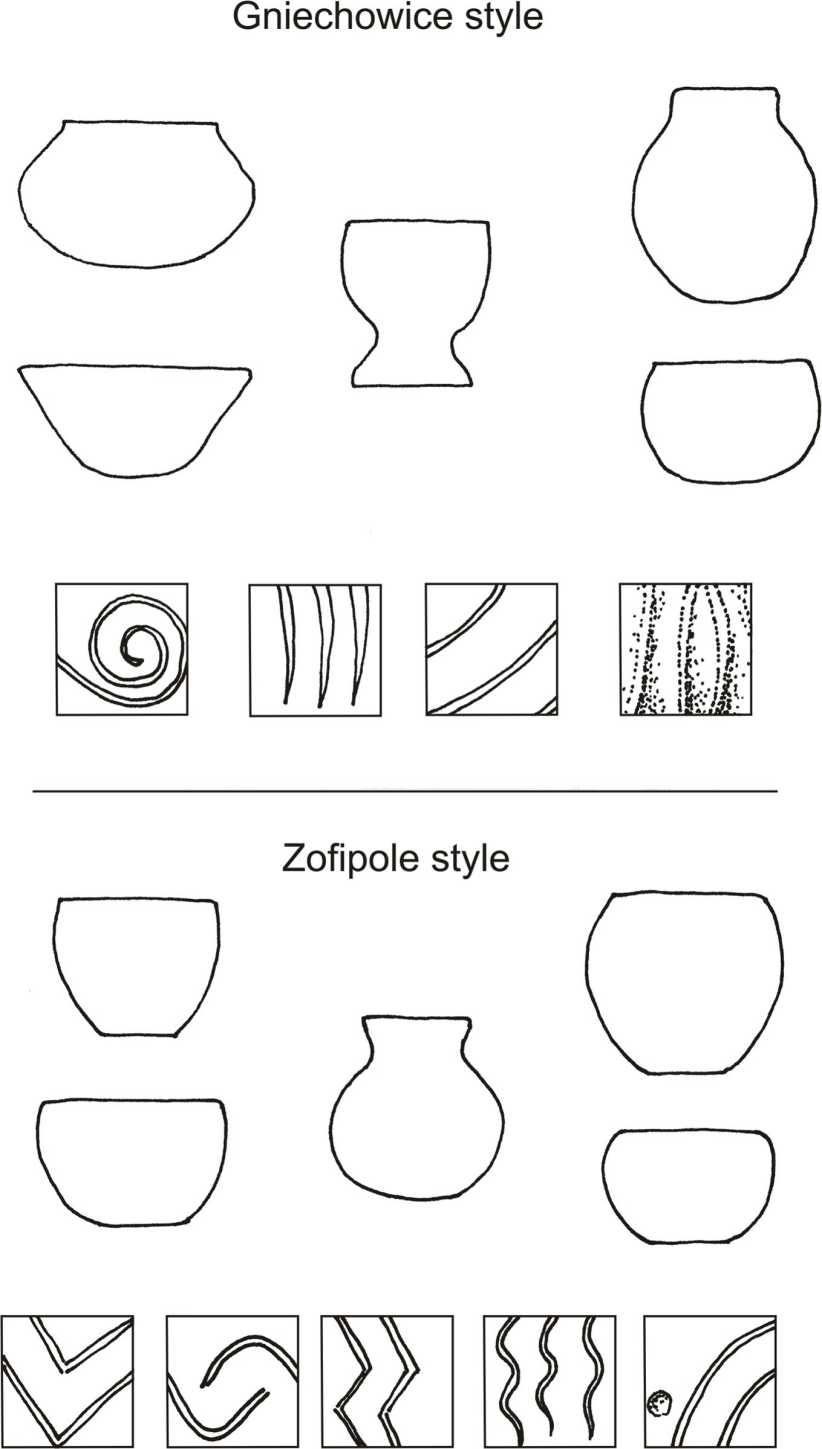
Gwoździec 2, Zakliczyn distr. **Vessel forms and decorative motifs from the I phase of LBK in Poland**.

**Fig 7:**
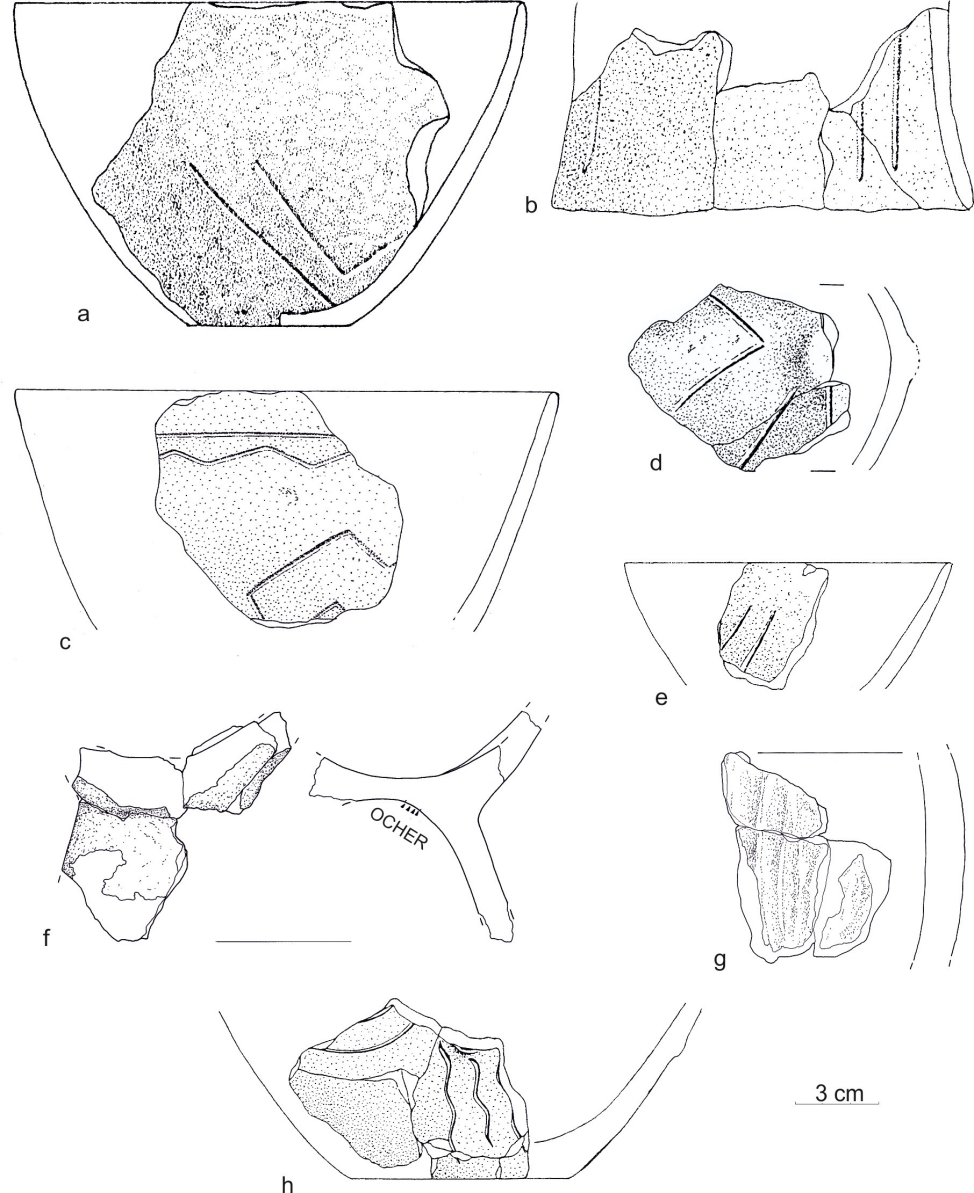
Gwoździec 2, Zakliczyn distr. **Selection of pottery connected with the I phase of LBK.** Materials: a, b, f–feature no. 1, c—feature no. 28, d, e, h–feature no. 23, g—feature no. 135.

From the settlement features dated to the old LBK phase, nearly six thousand pottery fragments were obtained. The minimum quantity of vessels, based on the number of rims and conjoined fragments, was estimated at ca. 300 specimens. The set of forms is typical of this chronological stage, including mainly: bowls with varying degrees of walls eversion, footed vessels, vessels with high necks, and globular vessels (Fig 6). The most characteristic feature of ornamentation is that of singular, wide, engraved lines (up to 4 mm wide). They form volutes, S-shaped, and simple geometric motifs, straight lines, or arcs. Larger vessels are decorated with knobs of various shapes, plastic bands with finger impressions, and with *barbotino* ornamentation (Fig 7). It should be noted that some of the pottery are characterized by ornaments typical of the oldest stylistic phase distinguished in Polish areas, i.e., Gniechowice (Ia), which occurs jointly in the same pits together with Zofipole (Ib) ceramics This is primarily *barbotino* and wide (2–4 mm), U-shaped engraved lines. Such ceramics make up about 30 percent at the site, and in individual features belonging to the building complex they range from 10 to 45 percent. The majority of pottery can be associated with the Zofipole (Ib) phase, which corresponds to the style of the Flomborn phase in Germany and the Ačkovy phase in the Czech Republic and Moravia [1]. Therefore, the Gniechowice elements can be considered in this context as archaic motifs. In addition, the same features have individual fragments with the initial motif of music note.

**Fig 8:**
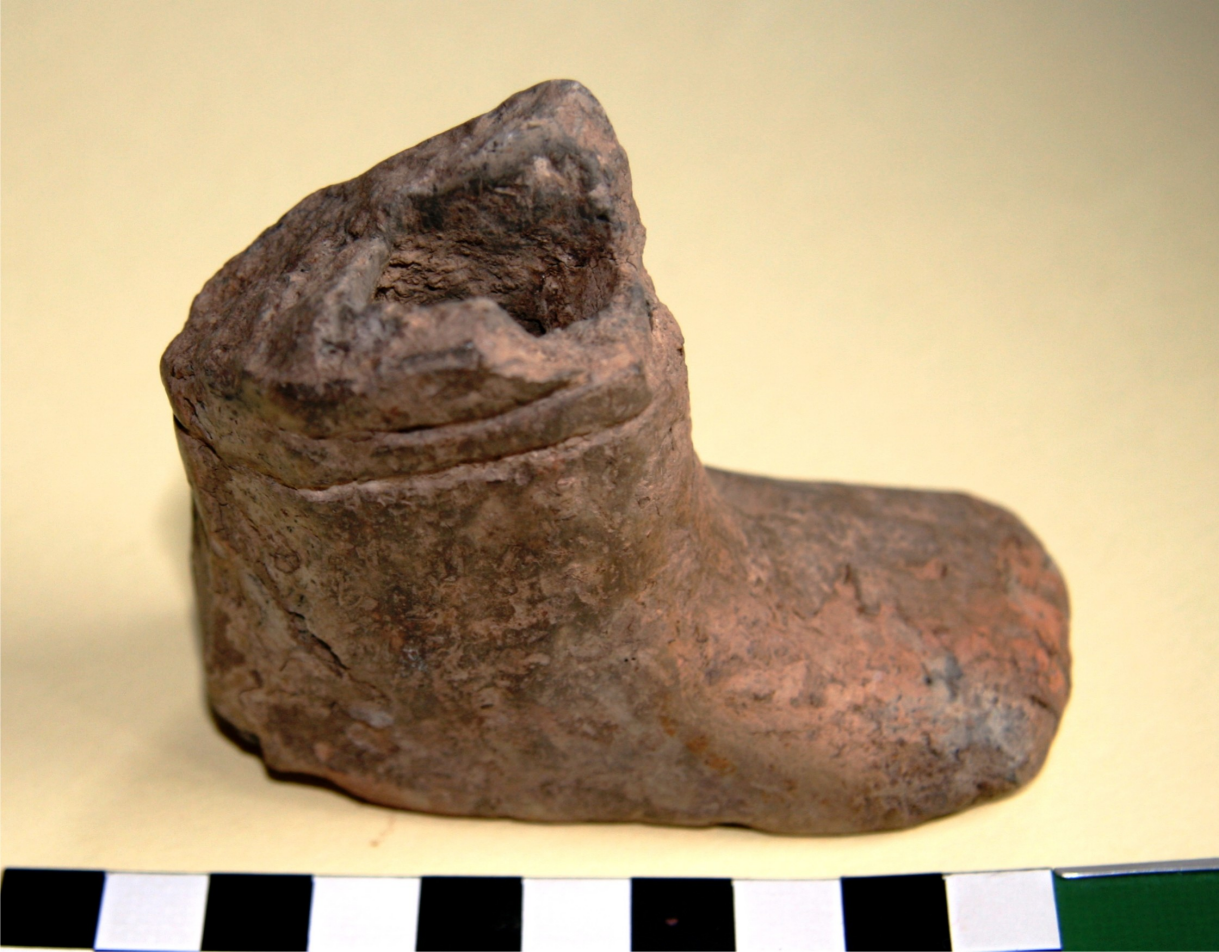
Gwoździec 2, Zakliczyn distr. **Fragment of anthropomorphic figurine (foot) from feat. No 23**.

Amongst the pottery noteworthy is also a unique find–namely, a fragment of an anthropomorphic figurine (Fig 8). It comes from feature no. 23, discovered near the eastern wall of the house. This is a fragment of a leg with a foot. The specimen is made in a realistic manner: individual toes were marked by means of short incisions. Above the ankle there are two horizontal, engraved lines (an image of decoration, a bracelet, maybe). The preserved fragment must have been a part of a quite large figure (the foot is ca. 9 cm long and 4 cm wide, while the preserved height amounts to 7 cm). A hole visible inside the artefact indicates that the figure was most likely formed on a wooden framework [10].

Within the pottery assemblage 19% of all specimens were thin-walled vessels, 33% medium-walled, while almost half (46%) were thick-walled vessels. A great majority of thin-walled pottery was made of clay materials, described here as fine ceramic fabric (87%). Only 13% of fine pottery was made of fabric with mineral grains content (type III) but most of the medium vessels were made of this type of fabric (51%). Medium pots were also made of fine fabric (35%) and fabric with an admixture of clay clasts– 14% (type IV). Coarse pottery (or storage vessels) was usually made of the fourth type of fabric (62%). Only 6% were made of fine fabric and 32% of fabric type III. Microscopic observations and analysis allowed the authors to specify the above-mentioned division.

In total 24 pottery fragments from both excavation seasons were subject to microscopic study (Table 1). They are dated to LBK phase Ia/Ib. Among them ten fragments come from fine vessels (open and spherical bowls), eleven from coarse pots (spherical bowls) and three from medium pottery (a spherical bowl, an open bowl, and a bowl on an hollow foot).

**Table 1:** Gwoździec 2, Zakliczyn distr. List of the samples to optical microscopy analysis.

Sample no	sample code	type of sample	morphological type	decoration	LBK phase/ chronology	inventory no	feature/ location	fine/ medium/ coarse	fabric type
1	Gwoz02	pottery	spherical bowl?	decorated	Ia/Ib	inv. 621	135/2017	fine	II
2	Gwoz03	pottery	?	decorated	Ia/Ib	inv. 622	135/2017	coarse	IV
3	Gwoz04	pottery	spherical bowl?	undecorated	Ia/Ib	inv. 748	120/2017	medium	I
4	Gwoz05	pottery	?	decorated	Ia/Ib	inv. 665	120/2017	fine	III
5	Gwoz06	pottery	open bowl?	decorated	Ia/Ib	inv. 660	135/2017	fine	I
6	Gwoz08	pottery	spherical bowl	undecorated	Ia/Ib	inv. 610	135/2017	coarse	IV
7	Gwoz09	pottery	bowl on hollow foot	undecorated	Ia/Ib	inv. 583	135/2017	medium	III
8	Gwoz10	pottery	spherical bowl?	undecorated	Ia/Ib	inv. 743	125/2017	coarse?	IV
9	Gwoz17	pottery	spherical bowl	decorated	Ia/Ib	inv. 740	125/2017	coarse	IV
10	Gwoz18	pottery	open bowl	decorated	Ia/Ib	inv. 613	135/2017	fine	I
11	Gwoz19	pottery	spherical bowl	undecorated	Ia/Ib	inv. 279	135/2017	coarse	IV
12	Gwoz20	pottery	spherical bowl?	undecorated	Ia/Ib	inv. 632	135/2017	fine	II
13	Gwo127	pottery	open bowl	decorated	Ia/Ib	*	31	medium	I
14	Gwo128	pottery	bowl	decorated	Ia/Ib	176/1	31	fine	I
15	Gwo129	pottery	spherical bowl	undecorated	Ia/Ib	76/1	31	coarse	IV
16	Gwo130	pottery	open bowl	decorated	Ia/Ib	218/01	31	fine	I
17	Gwo131	pottery	open bowl	decorated	Ia/Ib	218/01	31	fine	III
18	Gwo132	pottery	bowl	undecorated	Ia/Ib	239/01	31	coarse	IV
19	Gwo133	pottery	bowl	undecorated	Ia/Ib	76/01	31	coarse	IV
20	Gwo134	pottery	?	undecorated	Ia/Ib	218/01	31	coarse	IV
21	Gwo135	pottery	?	undecorated	Ia/Ib	31/96	1	coarse	IV
22	Gwo136	pottery	bowl	decorated	Ia/Ib	115/96	1	fine	I
23	Gwo137	pottery	?	decorated—barbotino	Ia/Ib	37/96	1	coarse	IV
24	Gwo138	pottery	?	decorated	Ia/Ib	110/96	cultural layer	fine	I
25	Sur35	Holocene alluvial clay	-	-	-	-	-	-	-
26	Sur36	Holocene alluvial clay	-	-	-	-	-	-	-
27	Sur37	Holocene alluvial clay	-	-	-	-	-	-	-
28	Sur38	Holocene alluvial clay	-	-	-	-	-	-	-
29	Sur39	Holocene alluvial clay	-	-	-	-	-	-	-
30	Sur40	Holocene alluvial clay	-	-	-	-	-	-	-

**Fig 9:**
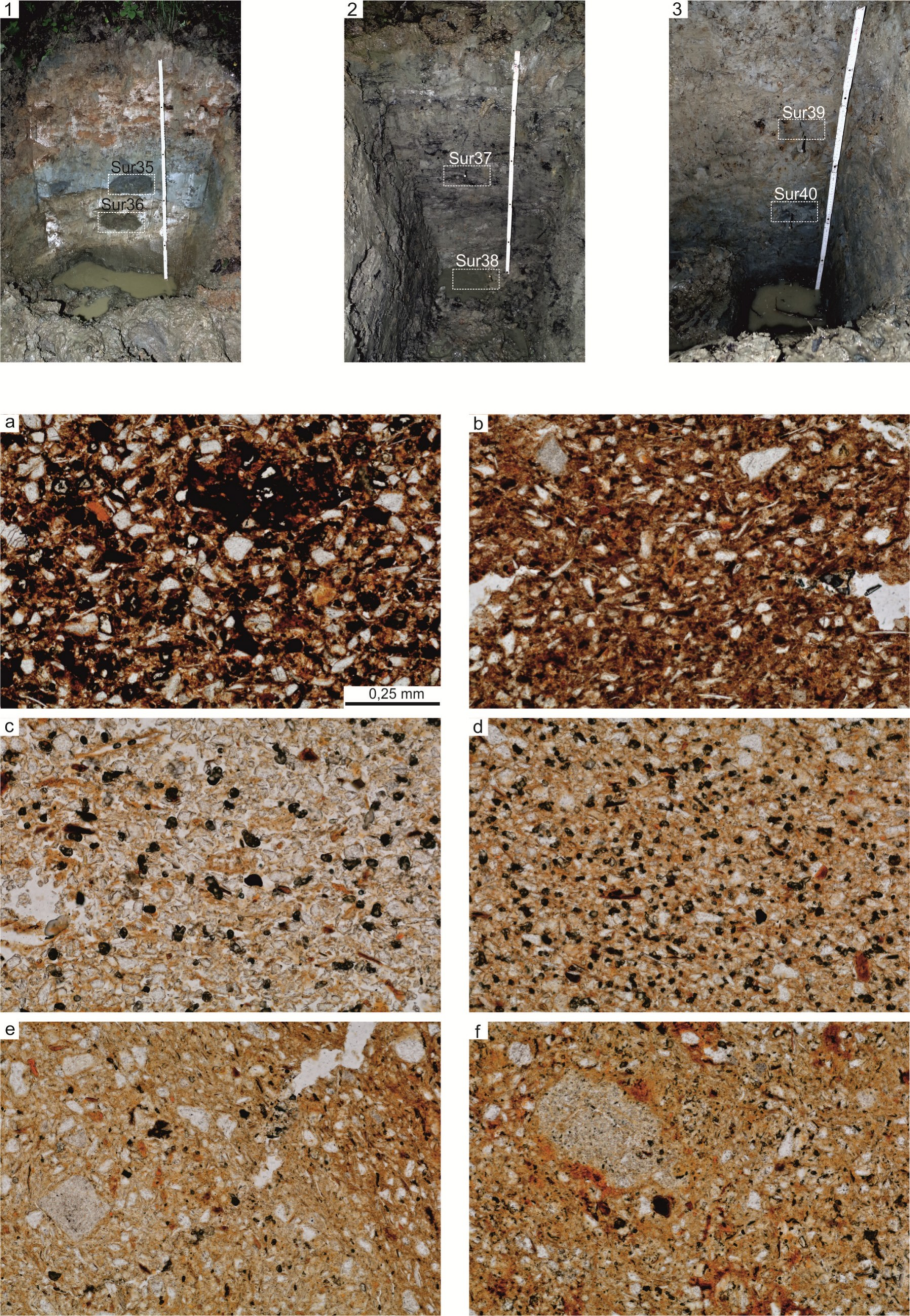
Gwoździec 2, Zakliczyn distr. **Testing of clay raw material.** Profiles of locations 1, 2 and 3 of ceramic raw material sampling (1–3); thin section microphotographs of ceramic raw materials (a-f); a–sample Sur35, depth approx. 0.6 m, clay rich in fine grains, iron oxide and hydroxides and fine mica flakes, PPL; b–sample Sur36, depth approx. 0.8 m, smaller content of silt fraction, orange color of clay matrix, PPL; c–sample37, depth approx. 0.6 m, high content of silt fraction and fine opaque minerals, PPL; d–sample Sur38, depth approx. 1.1 mm, smaller content of silt fraction, very fine grained clay, PPL; e–sample Sur39, depth approx. 0,6 m, medium sorted, fragments of sedimentary rocks occur, PPL; f–sample Sur40, depth approx. 0.8 m, clay with the smallest content of silt fraction, fragments of sedimentary rocks occur.

Furthermore, six samples of raw clay material were taken for comparison study. Three locations in the stream valley nearby the site were chosen for sampling clay material (Fig 9A–9F). From each location two samples were taken: from the upper and the bottom part of the profile.

Thin sections of potsherds were prepared for the study using a polarizing microscope. The quantitative microscopic analysis, involving point counting, allowed the authors to establish the percentage content of components such as clay minerals, quartz, potassium feldspars, plagioclases, muscovite, biotite, carbonates, sedimentary, igneous and metamorphic rock fragments, reused potsherds, as well as organic material. Thin sections were provided with petrographic descriptions. The descriptions included the percentage content of particular components, level of fabric kneading, as well as firing atmosphere and temperature. The data collected were then used for comparative analysis and provided the grounds for classification of the samples according to clay preparation and finished product firing technologies. Approximate firing temperature was determined based on thermal transformation of clay minerals, through observations of the degree of transformation into amorphous isotropic substance, as well as observations of biotite, hornblende and glauconite [11]. Grain sizes were measured under the polarizing microscope, using a micrometer scale. Percentage volumetric content of particular fractions was estimated using the planimetric method or quantitative microscopic analysis involving point counting. This study adopts the classification into grain fractions developed by the Soil Science Society of Poland [12].

### Flint artefacts

In the studied lithic assemblage we identified 9 cores and 4 flint chunks with single scars, 2 splintered pieces, 5 flint hammerstones, 270 flakes, 214 blades, 66 retouched tools and waste created during their preparation and 171 chips and chunks (Table 2). They were discovered within 28 features, despite the fact that during fieldwork wet sieving was used. The number of lithic artefacts discovered in features varied, and only in one feature (no. 10B) did their quantity reach the number of 150 finds.

**Table 2:** Gwoździec 2, Zakliczyn distr. General structure of the inventory and it context.

Feature	Thermal chunks	Cores	Flint hammerstones	Splintered pieces	Flakes	Blades	Chips and fragments	Retouched tools	Burin spalls	Total
1		1			30	22	25	4		82
2							1			1
7					5	6	8			19
8					4	4		2		10
10	1				12	12	4	3		32
10A'			1		11	8	9	2		31
10A'				1	18	21	10	11		61
10B		1			45	52	34	17	1	150
22			2		11	9	9	4		35
23					8	2	4	2		16
24			1		16	11	7	1		36
25					7	6	6	1		20
26					1	2				3
27					5	2				7
28					2	2	2			6
29.		1			42	23	21	6	1	94
30.		1			4	2	1			8
31.	2	2		1	37	15	19	6		82
32.		1	1		4	4	2			12
33.	1					1				2
64						1				1
88					1					1
90					1			1		2
96					2	3	4	2		11
110					1					1
120							2			2
125		1				1				2
129							1			1
130						3	1			4
133						1	1	1		3
135		1			3	1		1		6
Total	4	9	5	2	270	214	171	64	2	741

Raw materials used for production of lithic artefacts were identified based on the authors’ experience and comparative collection, stored at IAE PAS, ISEA PAS and at the Jagiellonian University in Kraków. All finds were classified into seven groups: cores, splintered pieces, hammerstones, flakes, blades/bladelets, retouched tools (formal tools), and chips and chunks. The next step was to record the metrical attributes (length, width, and thickness), as well as all descriptive features, such as: cortex presence, dorsal scar pattern and butt features. Descriptions of the retouched tools employed the criteria proposed by Ginter and Kozłowski [13] and Inizan et al. [14].

During excavations at the Gwoździec site, more than seven hundred stone artefacts related with the Gniechowice phase settlement were collected and use-wear analysis of them is underway. Here we present the results of microscopic observations of the artefacts discovered during the most recent excavations. The Olympus SZX9 stereomicroscope (up to 114×) was used for the initial selection. Chips and four flakes without any macroscopic traces were excluded from further observations. The Nikon ECLIPSE LV100 metallographic microscope (50–500×) was used for high-power analysis of twenty-two specimens. Prior to the microscopic observations, all the artefacts besides a sickle insert were cleaned in water, in an ultrasonic tank. The experimental collection gathered by one of the authors (B. K-D) was used as reference materials.

### Plant remains

Only materials from the oldest settlement phase have been considered in the article. For archaeobotanical investigations a total number of 123 samples of 338.1 litres in volume were taken systematically from excavated features, such as differing pits and postholes of the early LBK phase. The sampling method aimed at gathering the most representative quantity of plant material sufficient to meet the criterion of statistical reliability for each archaeological layer. These samples were floated in the laboratory of the Department of Palaeobotany of the W. Szafer Institute of Botany of the Polish Academy of Sciences (IB PAS), through sieves with mesh diameters of 0.5 mm and 1.00 mm. The obtained material was segregated and all plant remains were collected, such as fruits, seeds, vegetative parts of grasses, and wood charcoals.

All plant material, including charred and uncharred diasporas, was determined using comparative collections from the IB PAS and the Archaeobotanical Laboratory at the Institute of Archaeology and Ethnology of the Polish Academy of Sciences and specialist literature [15, 16, 17]. Examinations were carried out by means of a binocular microscope with 10–40 magnification.

Wood remains preserved in the form of charcoal were identified using a reflected light microscope with magnifications of 100, 200, and 500x. Moreover, a scanning electron microscope was employed at the Laboratory of Field Emission Scanning Electron Microscopy and Microanalysis at the Institute of Geological Sciences of the Jagiellonian University (Kraków Poland). Each charcoal fragment was broken along three anatomical wood sections: transverse, longitudinal radial, and longitudinal tangential. Taxonomical identifications were made by comparing the actual specimens with those published in the atlases of wood anatomy [18, 19]. For antracological analysis collections of the IB PAS were used. The rank of identification (species, genus, family, etc.) depends on the size, anatomical characteristics of the wood, and the state of preservation of charcoal fragments [18, 20]. However, species is mostly indicated if at least one species of a genus exists in local flora [21]. Taking into account the history of vegetation in Poland [22, 23], three taxa (*Abies alba*, *Carpinus betulus* and *Fagus sylvatica*) are considered to represent any possible contamination resulting from an occurrence of ice wedges. The fact of mixing deposits with these last-migrating trees has already been recorded in the related literature [24, 25, 26]. The samples containing these taxa were excluded from the analysis of the Neolithic charcoal assemblages. Due to a small number of charcoal fragments, all of them were analyzed and taxonomically identified. Apart from taxonomic identification, wood curvature was determined, if possible, in order to acquire the information about wood diameter used by prehistoric humans [27]. A presence of fungal hyphae and xylophagous insects was noted as well as evidences of their activity, which can suggest usage of dead wood [28].

Latin plant names were given according to *Flowering Plants and Pteridophytes of Poland*. *A checklist* [29].

In general, plant remains were found in a small number in individual samples gathered from the site at Gwoździec. The density of plant remains per 1 litre was determined as extremely low since less than one specimen (fruits and seeds) per litre was found. With regard to wood charcoals, 1,162 fragments were documented, which makes about 4 fragments per litre. Charcoal fragments were mainly small (less than 4 mm^3^) and poorly preserved, with their anatomical structure incrusted with sediment particles. A great majority of the examined plant material has preserved in a charred state (Tables 3–5). The uncharred plant remains found at the settlement (summary list in Table 6) are considered to be present-day contaminations due to unfavourable preservation conditions in features discovered in dry deposits [21].

**Table 3:** Gwoździec 2, Zakliczyn distr. Charred plant remains preserved in the pits.

taxa name	kind of remains	number of feature					
		1[30]	25[30]	120	125	135	total
*Triticum dicoccon*	c	217	16	1	2	3	239
*Triticum dicoccon*	g	6			1		7
*Triticum dicoccon*	sp	1		1			2
*Triticum monococcum*	c		1				1
*Triticum dicoccon* vel *T*. *monococcum*	g		1				1
*Triticum dicoccon* vel *T*. *monococcum*	sp		1				1
*Cerealia* indet.	c		55		4	8	67
*Bromus secalinus*	c					3	3
*Chenopodium* t. *album*	s				8		8
*Fallopia convolvulus*	f				1		1
*Polygonum aviculare*	f				2		2
*Bromus* sp.	c	4			4		8
Fabaceae indet.	s			2			2
*Carpinus betulus*	f			1			1
total		228	74	5	22	14	343

kind of remain: c–caryopsis, g–glume, sp–spikelet, s–seed, f–fruit

**Table 4:** Gwoździec 2, Zakliczyn distr. Charred plant remains preserved in the post holes.

taxa name	kind of remains	number of feature					total
		87	88	89	90	130	
*Triticum dicoccon*	c	5		1	1	4	11
*Triticum dicoccon*	g				1	1	2
*Triticum dicoccon* vel *T*. *monococcum*	c					1	1
*Cerealia* indet.	c	8	1	2	6	5	22
*Bromus hordeaceus*	c					1	1
*Chenopodium* t. *album*	s					1	1
*Fallopia convolvulus*	f					2	2
*Polygonum aviculare*	f					3	3
*Bromu*s sp.	c				3	3	6
total		13	1	3	11	21	49

as in Table 1

**Table 5:** Gwoździec 2, Zakliczyn distr. Charred plant remains preserved in the others settlement features.

taxa name	kind of remains	number of feature						total
		31 [31, 32]	92	96	116	119	133	
*Triticum dicoccon*	c	3	2			1		6
*Triticum dicoccon*	g		1			1		2
*Triticum dicoccon*	sp		1					1
*Triticum monococcum*	c	1						1
*Triticum monococcum*	g					1		1
*Cerealia* indet.	c		1	2	1	3	2	9
*Bromus hordeaceus*	c		1					1
*Bromus secalinus*	c	1						1
*Chenopodium* t. *album*	s					1		1
*Echinochloa crus-galli*	c					1		1
*Fallopia convolvulus*	f	1						1
*Lythrum salicaria*	s					1		1
*Bromu*s sp.	c					1		1
Poaceae indet.	c					1		1
Poaceae indet.	chaff					1		1
total		6	6	2	1	12	2	29

as in Table 1.

**Table 6:** Gwoździec 2, Zakliczyn distr. Uncharred plant remains found in different features and representing the contamination with modern material.

taxa name	kind of remains	number of feature										total
		28	31	67	84	87	96	109	124	129	130	
*Betula pendula*	f		1				1					2
*Carex* sp.	f						4					4
*Chenopodium* t. *album*	s			3			5		11			19
*Polygonum aviculare*	f						1		1		6	8
*Polygonum minus*	f	9										9
*Polygonum persicaria*	f						2					2
*Scleranthus annuus* vel *S*. *perennis*	f			1			1					2
*Setaria pumila*	sp				1	1						2
*Vicia angustifolia*	s									1		1
*Vicia tetrasperma*	s							1				1
*Viola arvensis*	f		1				1					2
total		9	2	4	1	1	15	1	12	1	6	52

## Results and discussion

### Settlement

As mentioned above, the settlement in Gwoździec is the second site of the old LBK phase in Poland that has been investigated on a large scale. Thanks to intensive studies, an enormous number of new data referring to this stage of the LBK development was gathered. Most importantly, the one of the few examples of an LBK building complex in Poland was discovered. From previous excavations only minor parts of a complex of this kind were known–namely, from Stary Zamek [33]. Houses of similar construction are known from Targowisko, discovered a decade ago. However, they are not strictly dated, and the style of the ceramics indicates that the settlement is slightly younger, though still within the Zofipole phase [7]. At Targowisko there is no pottery with archaic features of the Gniechowice style, and music note motifs are more common. Noteworthy is the fact that in the old phase houses of slightly different construction were built when compared with those from younger phases. The house construction from Gwoździec can be classified as type 2 from the LBK phase I according to Modderman’s typology for Bylany (which is used to classification LBK constructions in Central Europe) [34]. The house from Gwoździec represents a typical construction consisting of 5 rows of posts (including three internal rows of massive supporting posts). It had a strengthened southern part and a transverse corridor in the middle of the construction. Along the walls narrow groove-like pits ran beneath the roof eaves. Other accompanying pits were located nearby. From the south, next to the entrance, a storage pit was located (feature no. 1), as well as other pits filled with a great amount of pottery and flint artefacts. The presence of a post centrally located in the pit, as well as several smaller posts around, indicates the covering of the feature with a kind of roof. The most numerous findings of houses from this phase come from Bylany in the Czech Republic [34] and Brunn am Gebirge in Austria [35].

The settlement in Gwoździec seems to have expanded eastward; nearby the building complex more settlement features were recorded, ones partly dug into the cultural layer mentioned above. Their fillings contained fragments of vessels identical in style to those obtained from the area of the building complex, but also to pottery decorated with ornaments corresponding with the successive developmental stages of this cultural unit, namely Early Music-Note phase (phase II of the LBK), and further to the east–Early Želiezovce phase (phase III of the LBK; see Fig 3).

### Pottery

Microscopic analyses of pottery fragments revealed that they were made of local clay material, containing elements typical of the Carpathian flysch. These included: ferruginous shales, mudstones and sandstones. Moreover, a few small chunks of crystalline rocks, consisting mostly of quartz and feldspar, were identified. The clay substance is often of dark orange colour (when oxidizing firing was performed), which is caused by saturation of the clay mass with ferruginous compounds. Moreover, ceramic fabric often contains opaque- minerals, while the shape of some of them can indicate an occurrence of pyrites that had undergone thermal transformation. Microscopic observations of collected clay samples revealed that their composition is similar to ceramic fabric. The most similar, in terms of mineral content, granulation and structure, are the clay samples obtained from the lower layers (on a depth of ca. 1 m below the ground level). They are characterized by lower content of grains of silty fraction, when compared with samples taken from the upper parts of the profile, and intensely dark orange colour (Fig 9B, 9D and 9F). Within those samples the authors recorded an occurrence of chunks of ferruginous claystone, mudstones and, more rarely, sandstones. They are the same elements as in the LBK ceramic fragments under study.

Ceramic fabric contains clay minerals, and small amount of grains of silt fraction. Only few ceramic masses contain a slightly higher amount of silt fraction. Muscovite flakes are usually very small and few. Clastic inclusion are represented by quartz, potassium feldspars, and the above-mentioned fragments of crystalline, most likely igneous rocks, as well as crumbs of sedimentary rocks. As an intentional admixture, organic material was used, which in shape and inner structure indicates an occurrence of grasses mainly from their inflorescence bracts [36].

**Fig 10:**
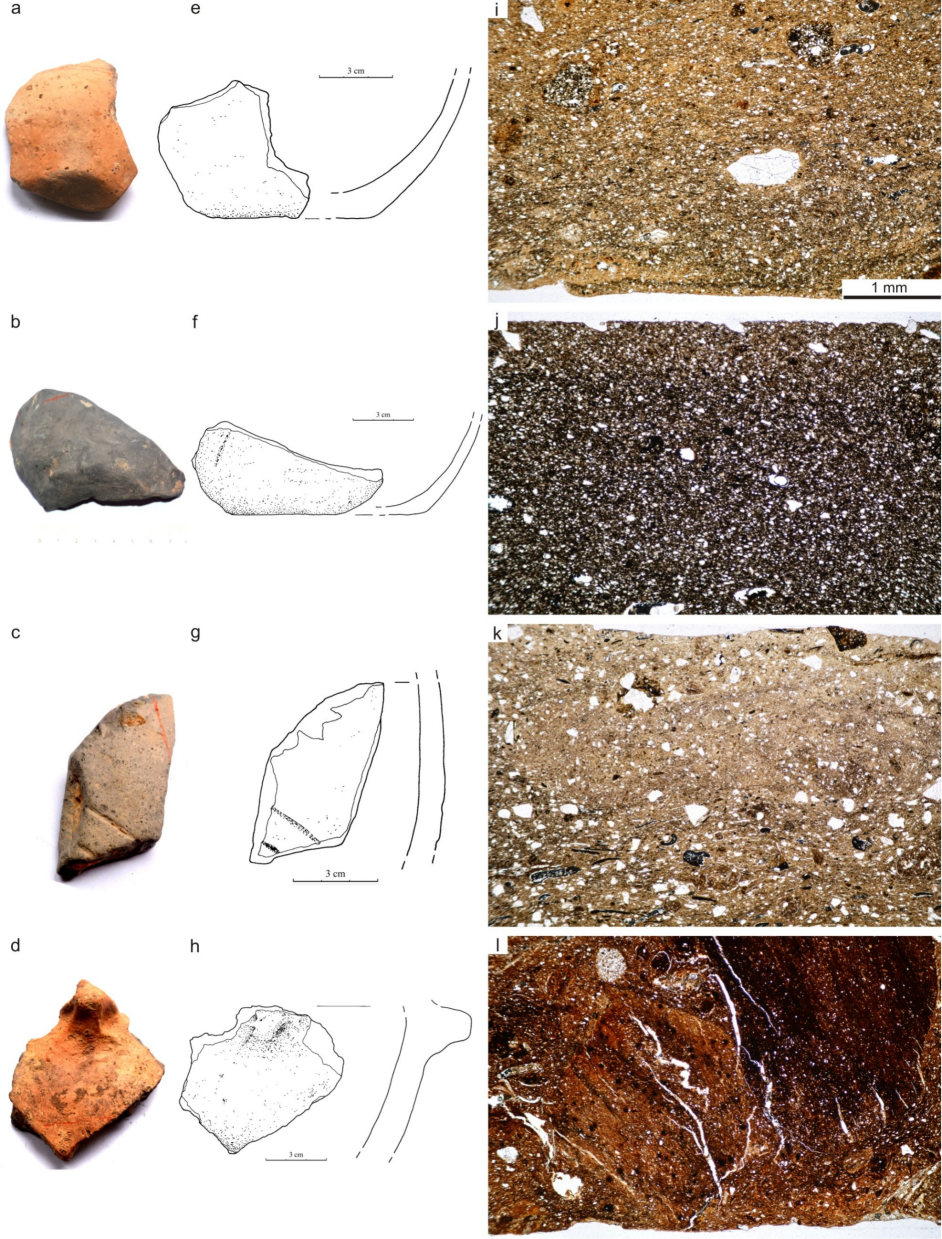
Gwoździec 2, Zakliczyn distr. **Testing of pottery.** Photography and drawings of selected fragments of vessels from each ceramic fabrics (a-h); thin section microphotographs of the ceramic vessels (i-l); i–sample Gwoz 4, a few sedimentary rocks fragments and crystalline grains occur in fine grained clay (type I), PPL; j–sample Gwoz 2, very fine grained, homogeneous, compact ceramic fabric (type II), PPL; k–sample Gwoz 5, fabric with mineral and organic admixture (type III), PPL; l–sample Gwoz 3, fabric with admixture of sedimentary rocks fragments and organic material (type IV), PPL.

Ceramic fabric shows a certain differentiation. Clay for production of fine ceramics was prepared in a different manner when compared with that used for manufacturing thick-walled vessels. Based on the results of previous investigations we identified several types of ceramic fabrics that characterized pottery dated to particular phases of LBK development in southern Poland [37, 38, 39, 40] (Table 7). Fragments of pottery from Gwoździec were classified into four ceramic fabric types. Fine ceramics (N = 5) were mainly made of fine-grained masses, moderately to well sorted. This fabric sometimes contained an organic temper and rarely unmixed clay clasts, as well as scarce chunks of sedimentary rocks. They were moderately or well mixed and compact (fabric type I; Fig 10A, 10E and 10I). Two fragments of thin-walled pottery were classified to ceramic fabric type II. They are very well sorted, with a high content of grains of silt fraction, and lacking intentional admixtures; this fabric is very homogenous and compact (Fig 10B, 10F and 10J). Another two fragments of fine pottery, decorated with engraved ornament, were made of ceramic fabric with higher content of thicker grains and organic admixture (type III; Fig 10C, 10G and 10K). Coarse vessels, also called storage pots, were produced using ceramic fabric containing greasy clay with fine-grained (silty) clastic material with an admixture of chunks of sedimentary rocks: ferruginous claystone, mudstones and sandstones. This fabric is characterized by a considerable content of intentional organic admixture (type IV; Fig 10D, 10H and 10L). Within ceramic fabric of this type are recorded quite numerous voids, being the remains of the burnout of organic material, which causes the higher porosity of this kind of pottery. Three vessels described as medium-walled (with plastic or engraved ornaments and smoothed surface) were manufactured using two types of ceramic fabric. Two vessels (sample 4 and 127: spherical bowl and open bowl, respectively) were made of ceramic fabric type I, whereas one specimen (sample 9—bowl on an hollow foot) of ceramic fabric type III.

**Table 7:** Gwoździec 2, Zakliczyn distr. Short description of ceramic fabric types and the number of pottery fragments with the division into fine, medium and coarse pottery.

LBK phase Ia/Ib	Fabric type I	Fabric type II	Fabric type III	Fabric type IV	Total
short description of ceramic fabrics	heavy clay, fine grained, moderately sorted, admixture of organic fragments, redox	silty clay, fine grained, well sorted, admixture of organic fragments; redox	heavy to silty clay, coarse grained, poorly sorted, admixture of organic fragments and sand; redox	heavy clay, fine grained, admixture of sedimentary rocks and organic fragments; ox	
fine vessels	6	2	2		10
medium vessels	2		1		3
coarse vessels				11	11
total					24

redox–reducing atmosphere with small access of oxygen, ox–oxidizing atmosphere

The physical properties of pottery produced using certain ceramic fabrics may vary. These differences resulted from the distribution and amount of crystalline grains, kneading of the clay mass and its porosity, as well as the temperature and method of firing. Ceramic fabric type IV, containing both organic compounds (that got partly or totally burnt out), as well as chunks of clay rocks (uncrushed clasts of clay) were fired in oxidizing atmosphere (with access of oxygen). Such conditions facilitate organic burnout, which causes the porosity of pottery. These ceramics gain hydroscopic properties, i.e., they are capable of absorbing moisture. This could have been an attractive property if the vessels were used for storing dry products (such containers prevent food from getting mouldy). The vessels in question are also capable of maintaining cooler temperature (thanks to a process known in modern physics as evaporative cooling). Whereas, pottery made of ceramic fabric type III, with a high content of mineral grains, could be more resistant to thermal shock, for instance, during cooking over an open hearth. Ceramic fabric type II is very well prepared and homogenous. Vessels made of such fabric may be described as fine quality specimens resistant to thermal shock. This is also noticeable in their appearance; their walls are smooth and compact, which may be perceived as an aesthetic value. Within the assemblage under study there were only two vessels made of this fabric (sample 2 and 20). Pottery manufactured using ceramic fabric type I is characterized by a poorer sorting of clastic material, less thorough kneading of clay, and significant prevalence of clay substance over the clastic compounds. Vessels made of this fabric may gain undesirable properties, such as increased abrasibility or susceptibility to damage caused by high temperatures. A great majority of pottery manufactured using ceramic fabric type I, II and III was fired under conditions of restricted access of oxygen. Temperatures of firing were estimated at ca. 700–800°C based on the observations of thermal transformations of some minerals.

### Flint artefacts

Almost the entire lithic assemblage was made of Jurassic flint from the Kraków region, imported to the site from a distance of about 70 km (N = 664, 89,6%). Some of those artefacts have natural surfaces covered by a thermal or a smoothed cortex. This demonstrates that part of this raw material was collected from secondary deposits, probably from the Vistula River valley and its smaller tributaries. A single flake from feature no. 27 was made of green radiolarite; the distal part of a blade discovered in feature no. 64 was made of grey-green chocolate flint, and two blades from feature no. 8 and 10A were made of erratic Cretaceous flint. The rest of the assemblage state heated artefacts most probably originally made of Jurassic flint (N = 73, 9,8%).

**Fig 11:**
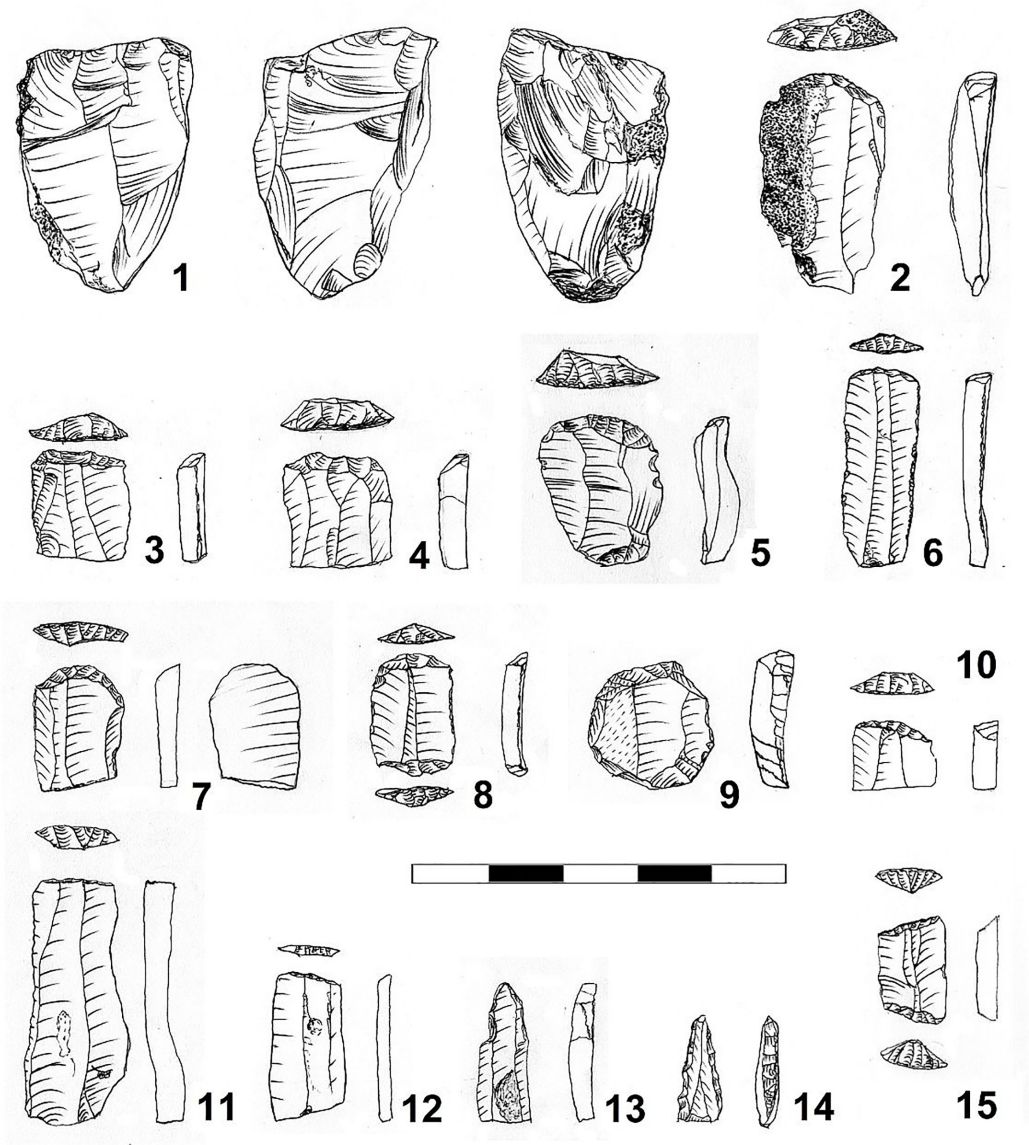
Gwoździec 2, Zakliczyn distr. **Flint artefacts.** 1 –core; 2–10 –endscrapers; 11, 12 –truncation blades; 13, 14 –perforators; 15 –trapeze.

In the Gniechowice phase assemblage, 9 cores and 4 flint chunks with single scars omitted in the further analysis were described. The finds represent all stages of core reduction including: tested nodules, initial cores, full debitage, and residual specimens. Two cores were transformed into hammerstones. The cores consist of a diverse group. Among them we can distinguish: five conical or sub-conical cores for blade (N = 1), (blade-flake (N = 3) and flake (N = 1) production (Fig 11:1), two flake cores with a changed orientation, single core made of flake and single semi-discoid core. The cores are characterized by an intensive preparation of the striking platform. They are small-sized, with the average dimensions not exceeding 7 cm. On some specimens relics of crests on the back and flaking surfaces are visible. Additionally two bi-directional splintered pieces made on flakes were identified.

From the Gwoździec site 5 hammerstones made of Jurassic flint were described. All of them were used for flint processing–most probably in the initial phase of decortication of the nodules.

The Gniechowice phase flint inventory contained 265 flakes. The flakes are dominated by specimens with a unidirectional dorsal pattern. Less frequent are flakes with a transversal or an oblique dorsal pattern; trimming flakes were sporadically noted. It is unclear if these specimens come from the initial phase of exploitation (some of them are covered by a natural surface) or if they were produced during changes to the orientation of the core, but it is likely that both possibilities took place. Because most flakes are non-cortical specimens, most probably they were made during exploitation and rejuvenation of cores.

Among the studied flint inventory, 214 blades were identified. We can distinguish only 16 complete specimens, with 86 proximal, 79 medial, and 33 distal fragments. The majority of the blades have no traces of a natural surface. As far as the dorsal pattern of the blades is concerned, specimens with a unidirectional scar pattern predominate, while some admixture of crested and secondary crested blades is present. Butts are mostly prepared by several blows, without any signs of butt abrasion. Moreover, there are no abrasive micro traces on the platform edge of cores. Amongst blades, half of all specimens bear marks of a lip.

Furthermore, 171 chips and small chunks of raw material were distinguished. They constitute thermal fragments of flakes and natural chunks and chips created during blank and tool production.

Amongst formal retouched tools (not including blades bearing traces of use) the authors identified 64 formal tools and additionally two burin spalls. Among them endscrapers (N = 32), truncated blades (N = 8) and perforator/borers (N = 7) prevail (Fig 11:2–15). Other tool groups occur much less frequently. Among them retouched blades (N = 4) and flakes (N = 4), trapezes (N = 2) and fragments of unidentified tools (N = 2) are the most numerous. Endscrapers are the most numerous tool group of this assemblage. The most frequent are specimens made on blades, although single flake endscrapers also occur. Among truncation blades, oblique, rather small specimens prevail. In the studied inventory, important groups include perforators made on slender blades, with a massive, carefully made working tip. The presence of minute trapezes is also significant, which, due to their size, were probably used as spear points and arrowheads elements.

**Fig 12:**
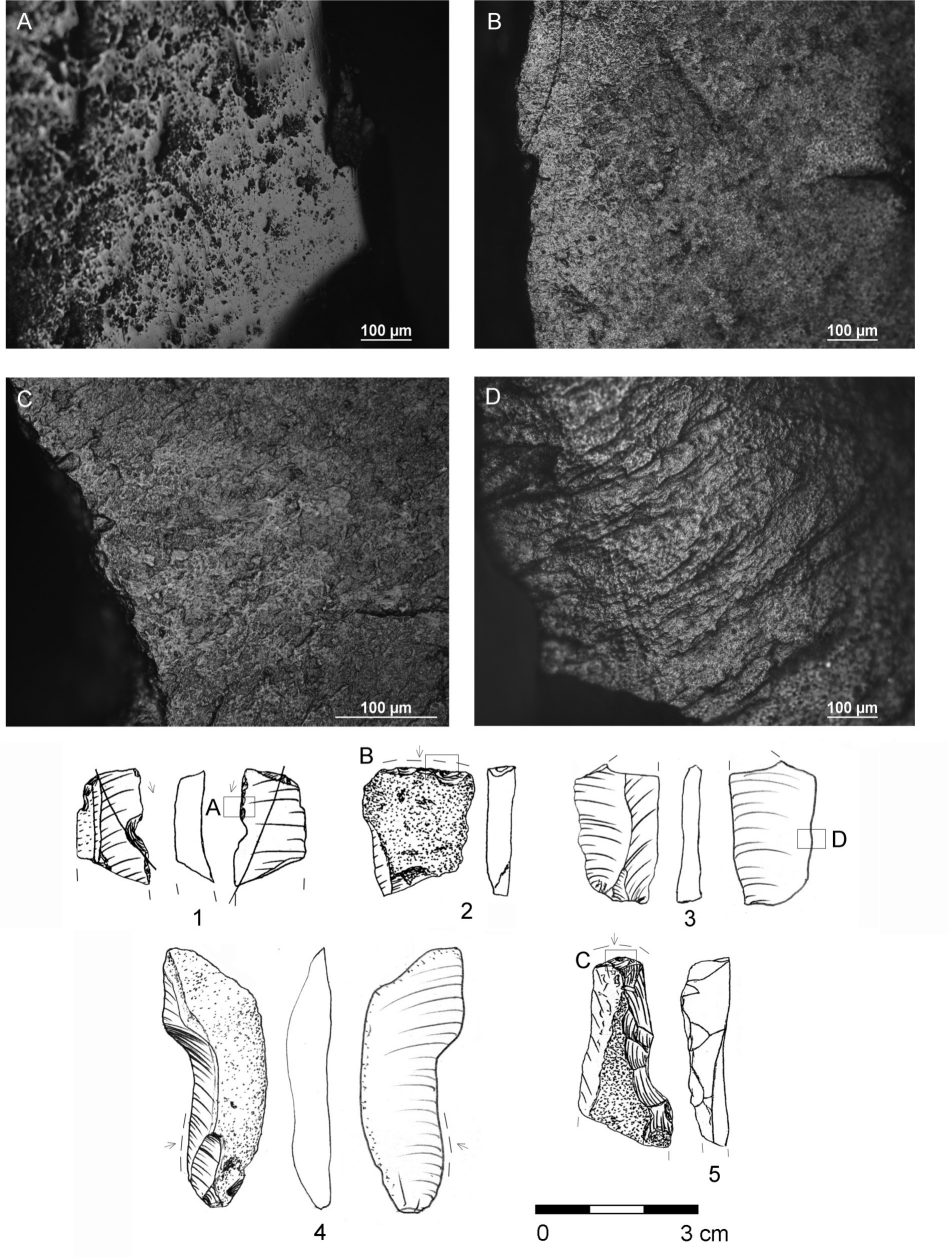
Gwoździec 2, Zakliczyn distr. **Lithic tools with traces of use.** Microphotographs **(**A-D) and drawings (1–5). Traces of: harvesting cereals (A, 1), scraping hide (B, 2), working hard material (C, 5), cutting wood or plants (4) and blade with worn out surface (D, 3).

Traces of use were recorded on four artefacts from the excavation seasons 2016–2018, including an atypical truncation blade, endscraper, a retouched blade, and a blade without retouch (Fig 12: 1–2, 4–5; Tables 8 and 9). Tools come from four different pits. The truncation blade (Fig 12: 1) was used for harvesting cereals and was inserted obliquely in a handle. This implement is a part of a cutting edge of a typical curved or L-shaped sickle used by LBK farmers, with one or more flint blades inserted at a low angle. Well developed, smooth sickle gloss covers more than one-third of the artefact in the area of the right distal protruding edge. The polish has an abrasive texture, with short striations and comet-shaped pits (Fig 12A:). The cutting edge was resharpened during its usage. The endscraper with a cortical dorsal surface (Fig 12: 2) was used for scraping fresh hide. It has a rounded working edge and a band of greasy polish along the edge (Fig 12B). The implement is broken and slightly heated, probably as a result of retooling. The retouched crested blade with a partially cortical dorsal surface (Fig 12: 5) was used for processing hard material, such as teeth. Wide patches of bright polish irregularly cover a part of the retouched edge (Fig 12C:). A complete blade, also with partially cortical dorsal surface (Fig 12: 4), was used for cutting wood or plants. The latter artefact, a broken blade (Fig 12: 3), was not used as a tool, but its surface and all the edges are worn out, probably as a result of long-lasting storage/transport (Fig 12D:). The cores, eight blades, flakes, chips and chunks bear no traces of use.

**Table 8:** Gwoździec 2, Zakliczyn distr. Results of usewear analysis.

Type	Traces of use	No traces	Total
Cores	-	2	2
Blades	2	8	10
Flakes	-	8	8
Chips and chunks	-	9	9
Total (wastes)	2	27	29
Endscrapers	1	-	1
Truncated tools	1	-	1
Retouched blades	1	-	1
Retouched chunks	-	1	1
Undetermined tools	-	1	1
Chisel	1	-	1
Total (tools)	4	2	6
Total (all)	6	29	35

**Table 9:** Gwoździec 2, Zakliczyn distr. Tool use.

Type	cereal	wood/plants	hide	hard material	other	Total
Endscrapers	-	-	1	-	-	1
Truncated tools	1	-	-	-	-	1
Retouched blades	-	-	-	1	-	1
Blades	-	1	-	-	1	2
Total	1	1	1	1	1	5

At site no. 2 in Gwoździec, 26 macrolithic stone finds were discovered. Amongst them there were 14 grindstone fragments, a single flake from a hammerstone, a single cube-shape stone with polished surfaces and two stones without preserved working edges or traces of use. Furthermore, 5 fragments of an adze/chisel and 3 flakes chipped of this kind of tools were identified (Table 10). All grindstone fragments were made of local sandstones. Local origin also has the flake knapped from a hammerstone made of quartzite. Only the adze/chisels were made of extra-local raw material, metabasites (also known in Polish literature as amphibolite slate), the outcrops of which are located in the Bohemian Massif. The implement is partially preserved. However, a hafting polish on the surfaces indicates that it was used as a functional tool.

**Table 10:** Gwoździec 2, Zakliczyn distr. Macrolithic stone inventory and it context.

Feature	Grindstone	Hammerstone flake	Undetermined stone artefacts	Chisel	Flakes from chisels	Unworked stones	Total
8				1			1
10A					1		1
10B				1	2		3
25	2						2
31				1	-		1
32				1	-		1
96	3	-	-	-	-	1	4
120	-	-	-	1	-	-	1
135	9	1	1	-	-	1	12
Total	14	1	1	5	3	2	26

### Plant remains

**Fig 13:**
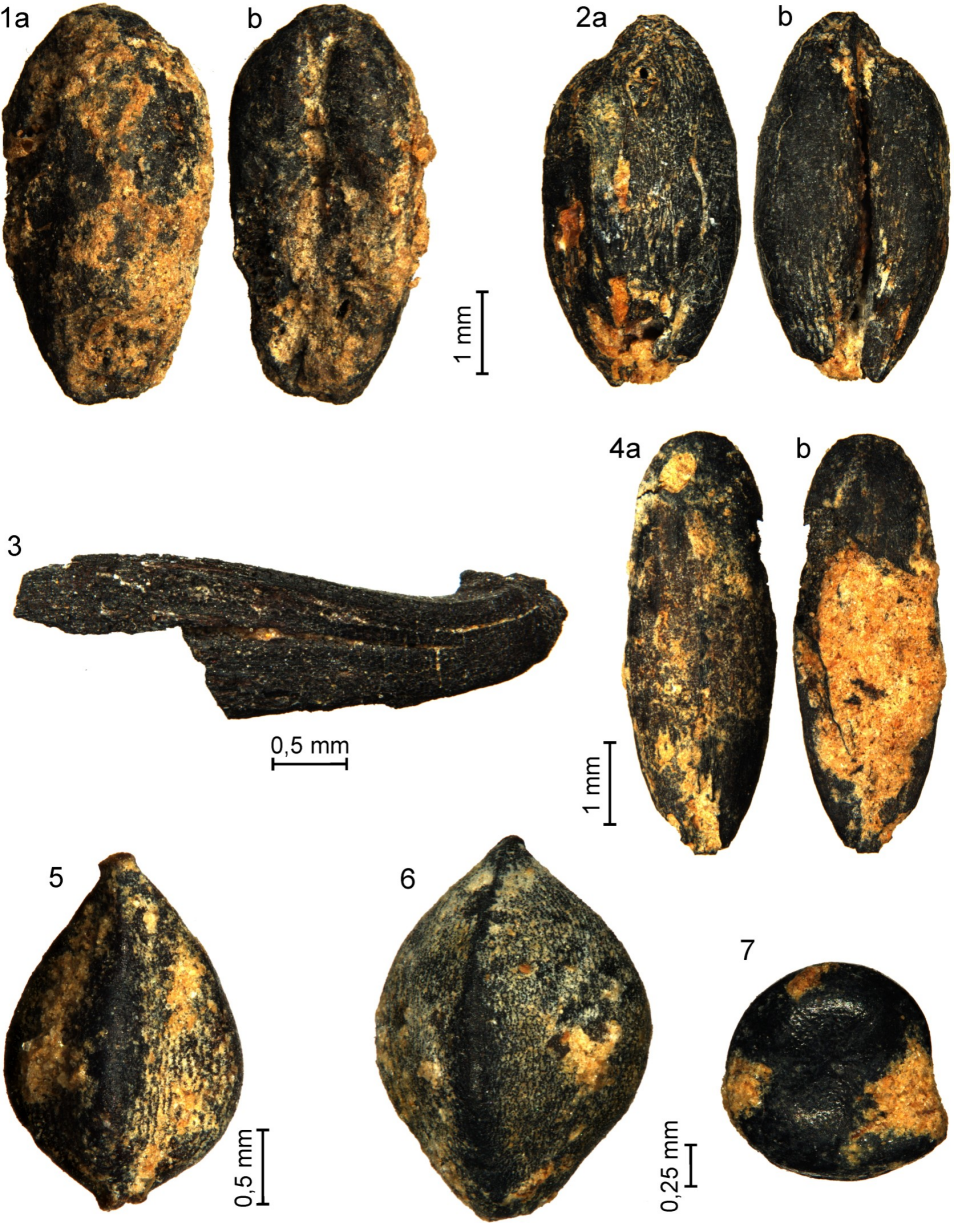
Gwoździec 2, Zakliczyn distr. **Plant remains.** 1 –*Triticum dicoccon*, one caryopsis from dorsal (1a) and ventral (1b) sides; 2 –*Triticum dicoccon*, one caryopsis from dorsal (2a) and ventral (2b) sides; 3 –*Triticum dicoccon*, glume; 4 –*Bromus secalinus*, one caryopsis from dorsal (4a) and ventral (4b) sides; 5 –*Polygonum aviculare*, fruit; 6 –*Fallopia convolvulus*, fruit; 7 –*Chenopodium* type *album*, seed.

Based on the charred plant material (seeds and fruit) preserved at archaeological site no. 2 in Gwoździec, dated to the LBK phase I, 13 taxa were identified, including 10 units determined to the level of species, one to the level of genus, and two to the level of family (Tables 1–3; Fig 13). A part of poorly preserved caryopses and fragments of spikelets were described as *Triticum dicoccon* vel *T*. *monococcum*, others were counted to the group of undetermined cereals *Cerealia* indet.

Emmer *Triticum dicoccon* Schrank prevailed among the identified cereals. It was followed by einkorn *Triticum mocococcum* L. The remains of hulled wheat included caryopses and fragments of spikelets. Most of the cereal remains were obtained from the pits (see Table 3), although they were also recorded in postholes (see Table 4) and other settlement features (see Table 5). Other cultivated plants were possibly represented only by unidentified seeds from the family Fabaceae.

A small number of wild herbaceous plant remains were found in the soil samples. Most of them represented field and ruderal weeds: *Bromus secalinus*, *B*. *hordeaceus*, *Fallopia convolvulus*, *Polygonum aviculare*, *Chenopodium album*, *Echinochloa crus-galli*, and *Bromus* sp. [41, 42, 43, 44, 45]. Wet meadow plants are represented by *Lythrum salicaria* [45].

An occurrence of fruit of *Carpinus betulus* (pit no. 120) requires a few words of comment. Hornbeam belongs to younger elements in Polish flora. According to palynological data, its origins in Polish territories is dated relatively late, to the end of the Atlantic Period, whereas the beginnings of its intensive expansion took place ca. 3500 radiocarbon years BP [22]. Probably this fruit is a younger or present-day contamination.

Amongst charcoal fragments 15 taxa identified to subfamily, genus, and species level (minimum number of taxa, M.N.T.) were documented (Table 11). These taxa represent both, conifers (gymnospermae) and broad-leaved groups (angiospermae). Amongst the conifers, three taxa were identified: juniper *Juniperus communis*, Scots pine *Pinus sylvestris* and spruce *Picea abies* or larch *Larix decidua*. A greater diversity of taxa is observed among broad-leaved trees and shrubs: dogwood *Cornus sanguinea*, hazel *Corylus avellana*, ash *Fraxinus excelsior*, maple *Acer* sp., alder *Alnus* sp., birch *Betula* sp., oak *Quercus* sp., willow *Salix* sp. or poplar *Populus* sp., lime *Tilia* sp., elm *Ulmus* sp., *Viburnum* sp. and Maloideae.

**Table 11:** Gwoździec 2, Zakliczyn distr. Results of antracological analysis from settlement features.

Taxa	number of feature											Total
	84	92	109	115	120	121	125	129	133	135	137	
*Corylus avellana*		9			12		8			32		61
*Fraxinus excelsior*		3	1		23	3	92	1	1	9		133
*Juniperus communis*									1			1
*Picea abies/Larix decidua*		2										2
*Pinus sylvestris*					1							1
*Acer* sp.	1	4		1			2			2		10
*Alnus* sp.									1			1
*Betula* sp.					5					2		7
*Cornus* sp.												0
*Quercus* sp.	7	33	4		55		52			192	18	361
*Tilia* sp.			2							2		4
*Salix* sp./*Populus* sp.		7										7
*Ulmus* sp.		1								4		5
*Viburnum* sp.					1							1
Betulaceae							1					1
Maloideae					4							4
Conifers		1	1		1		2					5
Broad-leaved		9	1		9	1	11		4	18	2	55
Sum of fragments	8	69	9	1	111	4	168	1	7	261	20	659
Bark		1										1
Indetermined: cf. bast of *Tilia* sp.												
Indetermined					2		6			4		12
Contamination: *Abies alba*					x			x				
Contamination: *Carpinus betulus*		x							x			
Contamination: *Fagus sylvatica*					x	x		x				

**Fig 14:**
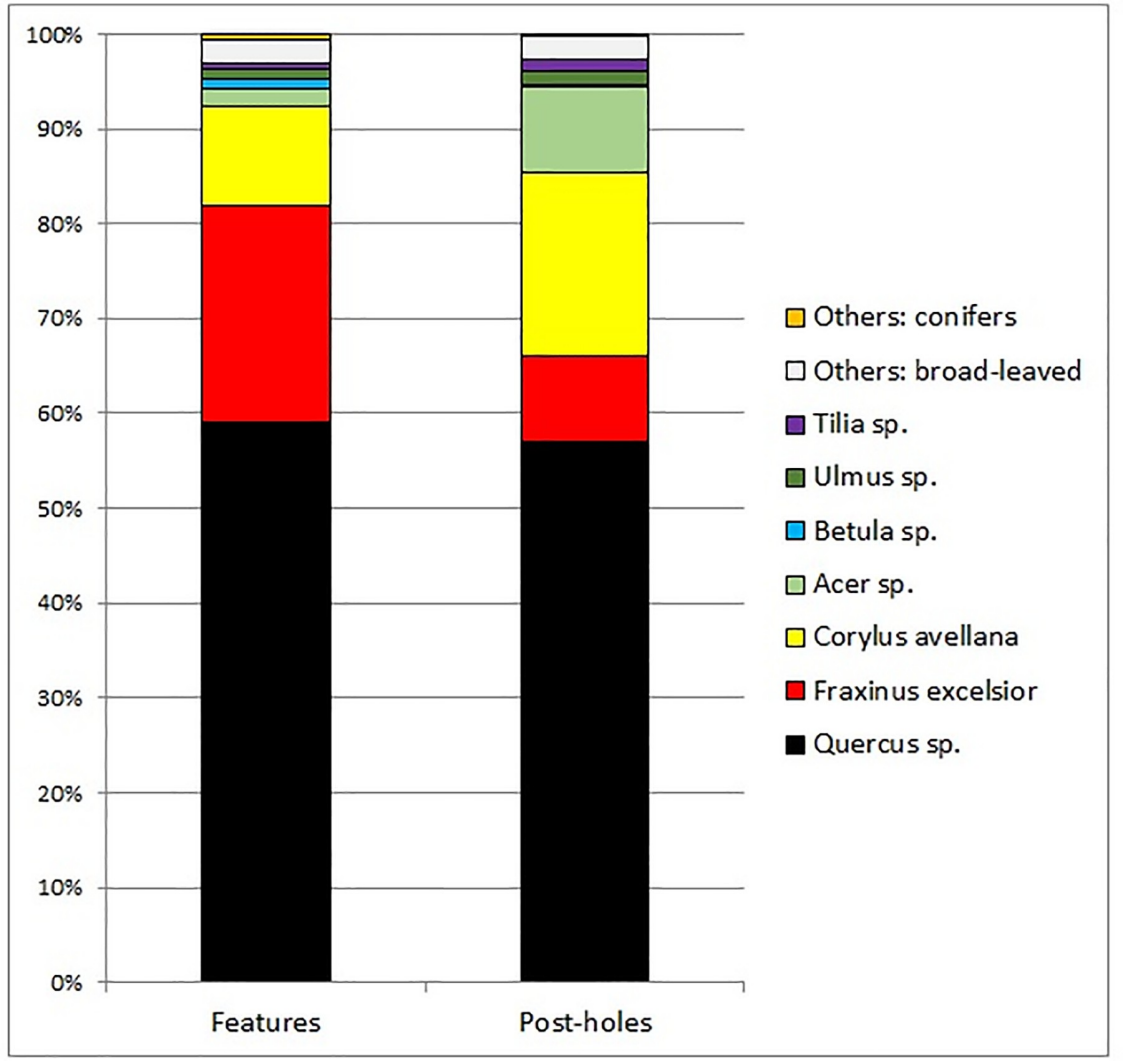
Gwoździec 2, Zakliczyn distr. Results of antracological analysis from post-holes.

Charcoal remains were found dispersed within sediments of archaeological features and postholes related with the house I (Table 12; Fig 14). In one case, remains of a burnt post were preserved (posthole no. 91), and wood of *Quercus* sp. was confirmed to have been used as construction material. In other postholes, charcoal fragments are very unlikely to represent posts, as they contained small fragments of taxonomically varied assemblages (M.N.T. is 10). A greater taxonomic diversity was recorded in the settlement features, where a total number of 15 taxa (M.N.T.) were documented. Remains of *Quercus* sp. prevail in both assemblages, from settlements features and postholes, exceeding 50% of a total number of charcoal fragments. *Quercus* sp. is followed by *Fraxinus excelsior* and *Corylus avellana*, but the first one is more frequent in settlement features, while the latter in postholes. Amongst other taxa, *Acer* sp. is the most widespread in both contexts, followed by *Ulmus* sp. and *Tilia* sp. The remaining taxa were found sporadically in a few or singular samples. Based on taxonomic diversity and a provenance of the charcoals from scattered fragments, it is very likely that these assemblages represent remains of fuelwood. This may also be confirmed by the presence of charcoals infected with fungal hyphae and bearing evidence of the activity of xylophagous insects.

**Table 12:** Gwoździec 2, Zakliczyn distr. Results of antracological analysis from post-holes.

Taxa	Post-holes								Total
	87	88	89	90	91	112	128	130	
*Corylus avellana*	3			43			1	33	80
*Fraxinus excelsior*				26	5			6	37
*Picea abies/Larix decidua*							1		1
*Acer* sp.	1			18	3		1	14	37
*Alnus* sp.				1					1
*Betula* sp.				1					1
*Cornus* sp.			1						1
*Quercus* sp.	5		17	61	107	2	1	42	235
*Tilia* sp.				2				3	5
*Ulmus* sp.				2				4	6
Betulaceae			1	2	1			4	8
Conifers		1	1	4	2		1	5	14
Broad-leaved			2	19	6			14	41
Sum of fragments	9	1	22	179	124	2	5	125	467
Indetermined: cf. bast of *Tilia* sp.				4					4
Indetermined				2				1	3

### Chronology

**Fig 15:**
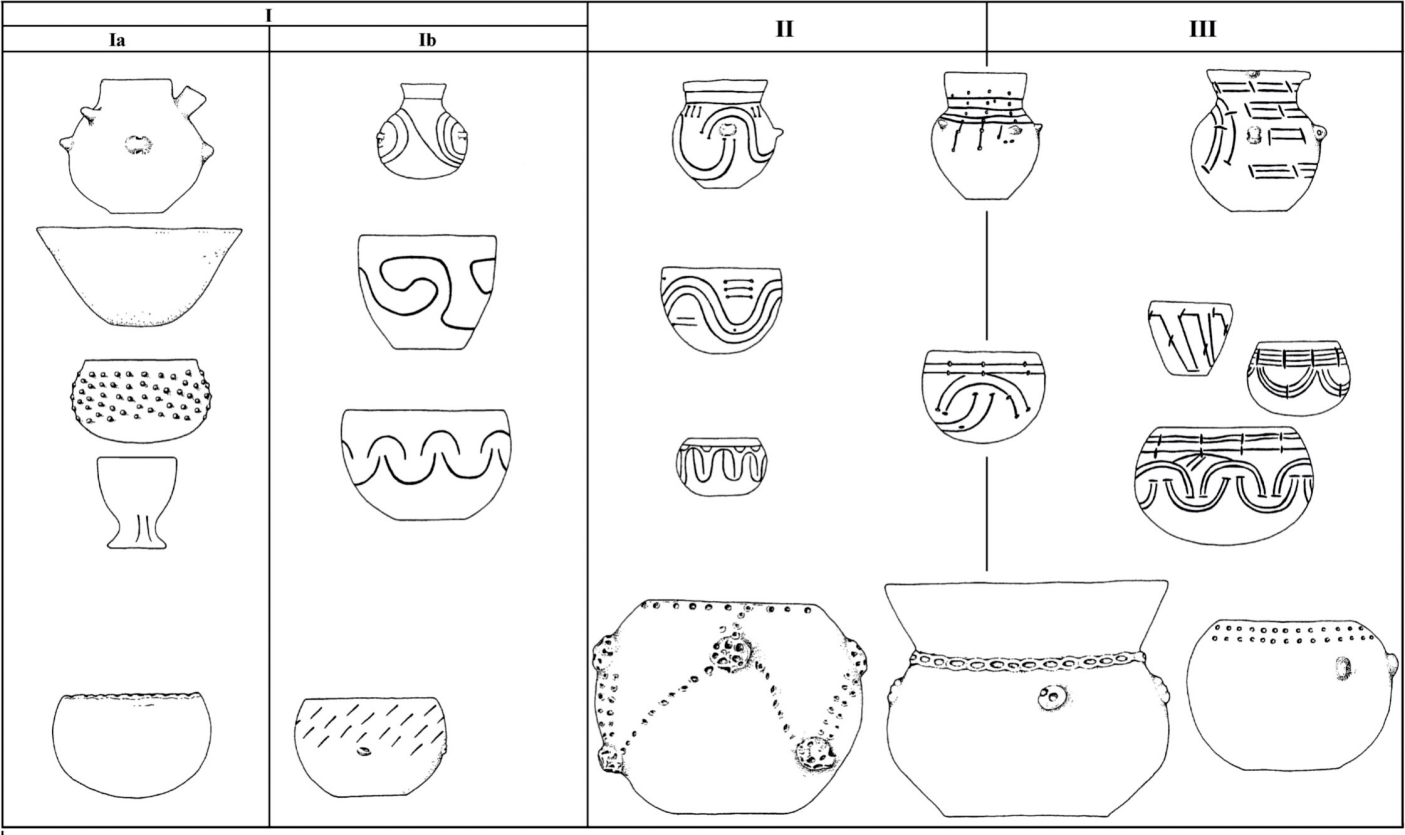
Pottery forms typical of the main chronological phases of the LBK on Polish territory.

**Fig 16:**
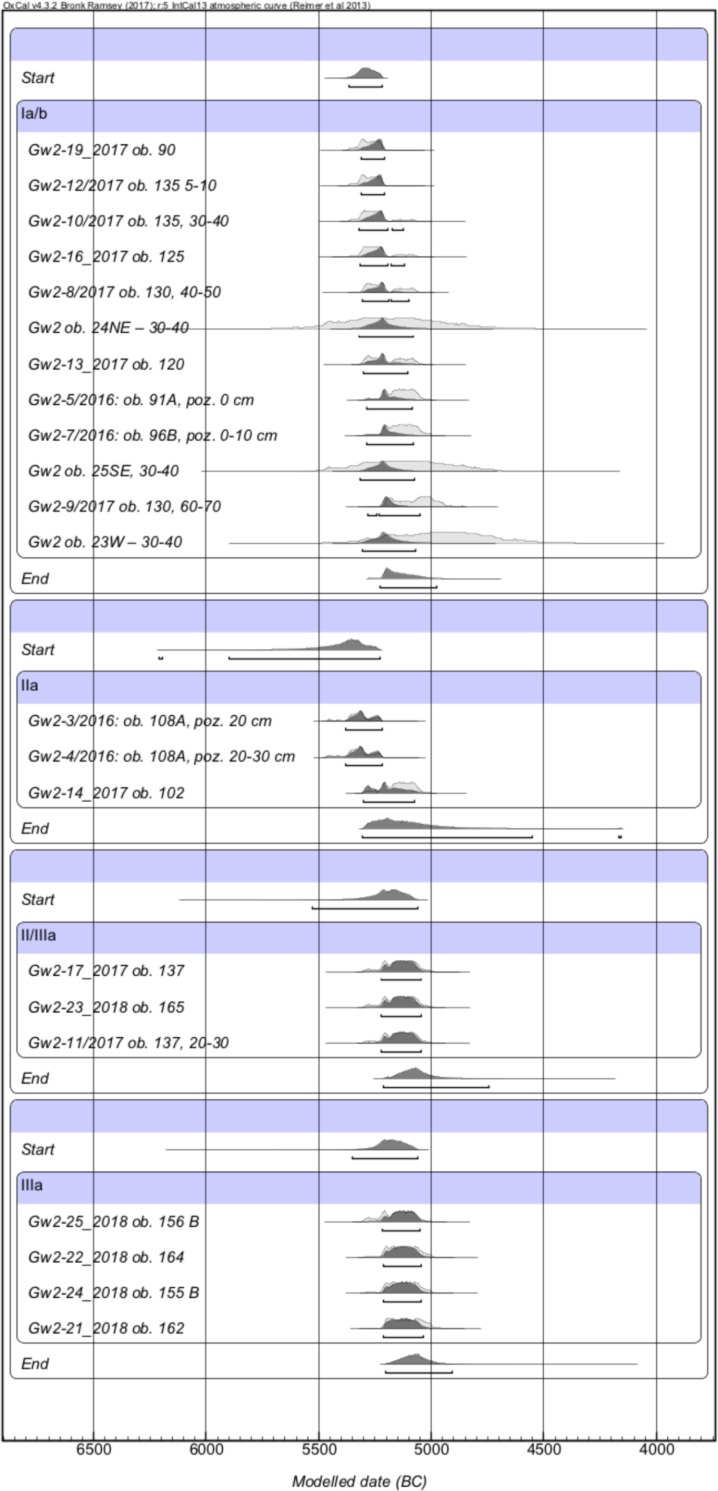
Gwoździec 2, Zakliczyn distr. **Diagram calibration of radiocarbon dating measurements** (dating by the Poznań Radiocarbon Laboratory).

The pottery stylistics of the early LBK phase shows the features of the Gniechowice (Ia) and the Zofipole subphases (Ib for the territory of Poland; Fig 15). This kind of decoration occurs over the entire area of the settlement. Therefore, in this particular case, the subphases Ia and Ib cannot be discussed separately; instead, they should be considered in general as phase I, namely the pre-Music Note phase. As mentioned above, the percentage of ornamental motifs indicates that the building complex should be entirely tied to the Zofipole phase. The presence of archaic ornamentation is characteristic for this period, and at the end of the phase we find the appearance of the initial music note motifs. This situation is visible for example at the already mentioned site in Targowisko 12/13 [7]. This is also in general consistent with the theory formulated by A. Kulczycka-Leciejewiczowa [46, 47], who stated that the LBK expansion into the area of Poland falls at the second half of phase I, namely the Zofipole subphase. However, in Gwoździec this process got even more delayed. From all of the above-mentioned zones of the settlement absolute datings were obtained. In total twenty-six 14C datings were performed (Fig 16). The samples included charred remains of seeds and charcoal, which were also subject to archaeobotanical analysis. Confrontation of these dates turned out to be surprising. They indicate that the settlement was occupied between ca. 5350/5300 and 5100/5050 BC, so in general to the period corresponding to the Music Note phase (II) [1, 48]. Typology and ornamentation of pottery from phase I would suggest that the beginning of the settlement was rather within the timeframes between 5400 and 5300 BC [49, 50]. The combination of these two facts indicates some developmental backwardness in Gwoździec.

## Conclusions

In the course of the investigations extremely interesting results of interdisciplinary analysis were obtained. Studies on the pottery indicate that the agricultural community of this settlement possessed the knowledge and abilities to produce technologically diversified ceramic vessels, characterized by various physical properties. These properties were induced intentionally, which may evidence a strongly rooted tradition of pottery production. The vessels were designated, probably already at the conceptual stage, for particular purposes, while the potters’ prowess allowed them to produce the desired products. Microscopic and technological analyses of the pottery are of special importance due to the fact that they were conducted based on a closed assemblage captured in a single house. Thanks to this it was possible to trace a building complex pottery production process.

As mentioned above, the most frequently used clay recipes in the early LBK phase at the site in Gwoździec was ceramic fabric type IV for coarse pottery and ceramic fabric type I for fine ceramics. Most likely they were not designed for cooking; instead they had other functions, such as storage vessels (food supplies) and other containers. In the assemblage under study less common types of ceramic fabric were those resistant to thermal shock (type II and III), with a significant content of crystalline elements (silt or sand). The percentage of vessels made of these types of ceramic fabric significantly increased in the successive stages of the LBK development in southern Poland. In the Music Note phase pottery produced using fabric type III, with mineral admixture, was particularly popular [40]. Conversely, in the latest LBK phase in southern Poland the percentage of pottery made of ceramic fabric type II increased, and was represented by homogenous, fine-grained vessels [40]. The changes seen in the Music Note phase should probably be connected with the inner developmental dynamics within the LBK. However, in the youngest developmental phase important external influences can be recorded, ones coming from cultural units of the Eastern Linear Circle (such as the Bükk Culture from the Carpathian Basin), shown, for example, by an inflow of products from the south, namely the territory of present Slovakia. Those influences could have affected some customs and techniques employed in ceramic production. This is indicated, for instance, by an emergence of pottery made of ceramic fabric with an admixture of chamotte. This kind of ceramics was common in the Bükk Culture [40, 51].

The described lithic assemblage from the site in Gwoździec sheds some light upon the subsistence strategies of the early phase of the LBK settlement and the expansion of early farming communities over the Polish Carpathian Foothills. From this area, except for Gwoździec, only a few much later LBK sites are known, ones also containing scarce lithic assemblages [4, 52, 53, 54, 55]. A similar situation is observed in south-western Poland [56, 57, 58, 59].

Raw material originally brought to the site in the form of natural nodules is not very numerous. Within 28 features 741 different types of artefacts were discovered. An occurrence of this raw material is typical of the early farming societies having inhabited southern Poland e.g., the area of Lesser Poland, Upper Silesia, and Sandomierz region, with examples of long-distance transport reaching the Czech Republic (Vedrovice), Austria (Kleinhadersdorf), Slovakia, and Hungary [60, 61, 62, 63]. Additionally, the existence of a single artefact made of green radiolarite and grey-green chocolate flint (providing that it is not an intrusion from the younger settlement phase, or artefacts obtained from the secondary outcrops) may evidence contacts with the Pieniny Klippen Belt area and Holy Cross Mountains regions, distant from the site about 100 km to the south and 150 km to the north. Long-range transport is confirmed only for an adze/chisel tool made of an amphibolite slate, transported from a distance of ca. 300 km. The occurrence of this kind of artefacts is not unique, as finds of this type are found in Poland within the entire extent of the LBK settlement [4, 64]. Production of flint artefacts took place directly at the site, which is proved by the presence of cores, hammerstones, flakes, and blades. The site itself could have been supplied either in the course of trips made by inhabitants to the source areas or by an exchange with other human groups. Although this question cannot be unequivocally answered, based on archaeological data. Preparation of cores was most often limited to the platform, but occasionally also to the flaking surface or the back of cores. Inferring from retouched tools, single platform cores for blades were most common. They were used for detaching laminar blanks, most suitable for tool production, but probably because of intensive flint use carried on the site, they are almost missed in this inventory. During this process single-platform blade cores were transformed into cores with changed orientation and at the final stage of reduction even on semi-discoid cores. Retouched tools, produced mainly on blades, clearly show that these blanks were preferred in tool production. That is why the presence of cores for flake could be interpreted as an effect of maximal exploitation of available raw material, connected with the intensive flint use carried out at the site. This supposition could be confirmed also by the presence of the cores created on a flint hammerstones.

The retouched tool inventory is much diversified, not standardized or specialized, both from a typological and functional viewpoint. There are few tools used for food production and crafting, such as harvesting cereals, processing of hide, bone/teeth, and wood or plants. However, some criteria employed for blank selection are noticeable. Functional tools were made on thick, irregular blades or flakes, including technical wastes, with complete or partial cortical or natural dorsal surfaces. Due to this the authors conclude that tool production was carried out rather on a limited scale and for direct building complex usage, which is reflected by traces of use.

Plant remains related to cultivation belong only to hulled wheat emmer *Triticum dicoccon* and einkorn *T*. *monococcum*. Both of these wheat species predominated in Early Neolithic agriculture in Poland [31, 65, 66, 67] and other European regions [26, 68, 69, 70, 71, 72]. The two wheat species were probably cultivated in the same fields in a mixture, as they have similar edaphic requirements and similar life cycles [21]. The presence of cereals confirms the agricultural nature of the first Neolithic settlement.

Plant assemblages coming from pits around the western, northern and eastern part of the house [31, 32, 33, 45] are quite scattered and not very numerous. This may result from the fact that there was a special zone of plants’ utilization in the southern area of the house I. The most frequent taxa of trees and shrubs are oak, ash, and hazel, which may suggest that they were indeed abundant in the settlement’s proximity if we assume that the most common categories are usually found more easily in the archaeological assemblage [73, 74]. Taxonomic composition and quantity proportions of charcoals indicate the existence of mixed deciduous woodland, more likely mid-Atlantic oak forest communities with hazel in a shrub layer. Contribution of *Corylus avellana* reached high values in the Carpathian Mountains in the 6^th^ millennium BC [75]. This species was also very frequently found in the charcoal assemblage from the late LBK site in Żerków [76], proving the importance of *C*. *avellana* in forests of the Carpathians in the Atlantic Period. In oak forests, usually of open canopy, trees of *Tilia* and *Acer* could have occurred. The different light demanding species belonging to the Maloideae subfamily could have grown as undergrowth or at the forest edge. Trees of *Fraxinus excelsior* and *Ulmus* could have mostly appeared in wet and shady zones with permanent good water supply, whereas *Alnus*, *Salix* and *Populus* in riverine and/or marshy areas. Ubiquitous and light demanding species of *Betula* and *Pinus sylvestris* could have also occurred within these woodlands. Taking into account the potential natural vegetation, oak woods and oak-hornbeam forests could have grown in this region [77], which may indicate that deciduous woodlands might have had fostering conditions for their development in local areas characterized by rich soils developed on loess substratum. However, it is very unlikely that typical oak-hornbeam forests had formed in the mid-Atlantic Period based on the absence of *Carpinus betulus*, and it is possible that these oak-dominated forests were similar to modern oak woods or they have no modern analogue at all, as in the case of the charcoal assemblage from Żerków mentioned above [76].

During the earliest stage of the LBK development (5500–5400 BC), an expansion of the agrarian population from the south covered primarily the areas of loess uplands in the Upper Odra and Vistula River basins, i.e., Lower and Upper Silesia and Lesser Poland. Towards the end of this phase, the expansion reached the north, to Kuyavia and Chełmno Land. It seems, however, that this stage of migration was merely an initial phase, a kind of “reconnaissance”, so to speak. In fact, from this period (Ia) ca. 30 sites are known from the entirety of Polish lands. Moreover, only some of them can be considered permanent settlements. Indisputable remains of long-lasting constructions in a form of post frame houses have been known only from one site in Stary Zamek in Lower Silesia [33], and now from Gwoździec. However, in many cases the sites dating back to the earliest phase can be interpreted simply as traces of campsites. Amongst them the most typical were found in the Kraków-Częstochowa Jura, where the traces of temporary stay of this population were recorded in a few Jurassic caves (Maszycka Cave and Okopy Wielka Dolna Cave) [5, 6]. Therefore, the first phase of migration of the agrarian population from the south is believed to have been associated with the exploration of new territories prior to permanent colonization and establishing permanent settlements. Further spread and aggregation of the settlement took place around the end of the LBK phase I, reaching its maximum intensity in the Middle-Phase II (Music Note phase) of this culture.

Due to this, the data gathered together in the course of the investigations carried out in Gwoździec is of great significance to studies on the dating, settlement, and economy of the early LBK phase in Poland. It should be explicitly noted that the "early phase" of LBK on Polish territory corresponds to the Flomborn/Ačkovy phase [78]. The next—middle phase—the Music Note phase, in southeastern Poland is absolutely dated to ca. 5300–5000 BC [1, 5]. This "overlapping" of dating from Gwoździec for the Gniechowice/Zofipole phase with general dating for the Music Note phase is surprising and creates some questions.

First of all the specific development of ornamentation during the early phase LBK is confronted with absolute dates. There is also a problem of stylistic backwardness regarding the Music Note phase. The building complex stylistically corresponds to the Zofipole (Ib)-Flomborn-Ačkovy phase, but the radiocarbon dates are not in accordance with the expected age of the discovered structures and pottery fragments. So in Gwoździec the Zofipole style was continued, while in other areas the Music Note phase developed. It is not known whether this was similar in Targowisko, as there are no 14C datings. This raises the question whether the site in Gwoździec is a special case, where we can observe a stylistic development from phase I to the beginning of phase III within a short period of time, corresponding, in terms of absolute chronology, with the Music Note phase of the LBK. Moreover, was this archaization in stylistics due to a certain location of the site, being slightly remote from the settlement centres extending to the south, towards the sub-mountainous regions? From south-eastern Poland, apart from the site in Gwoździec, the only 14C dates for the old LBK phase are known from Samborzec [3]. They are within the same timeframes of absolute dating. Due to a lack of dating (for example, for the Polish Lowlands only three 14C dates are known, burdened additionally with a huge statistic error) [79], it is difficult to compare the beginnings of the LBK settlement with other regions of Poland. It seems, however, that this process was younger than it has been assumed [5, 6], and that it took place not earlier than ca. 5350 BC. Nevertheless, there is a possibility of earlier, occasional presences of the LBK pioneers in caves of the Kraków-Częstochowa Jura. In fact, a few of them delivered the “pure (homogenous) materials” of the Gniechowice phase (Ia) of the LBK development, which are correlated with the Bíňa-Bicske LBK phase (ca. 5500/5400 BC) [1]. This issue, however, requires further dating, analyses and studies.
